# Active RNA Polymerases: Mobile or Immobile Molecular Machines?

**DOI:** 10.1371/journal.pbio.1000419

**Published:** 2010-07-13

**Authors:** Argyris Papantonis, Joshua D. Larkin, Youichiro Wada, Yoshihiro Ohta, Sigeo Ihara, Tatsuhiko Kodama, Peter R. Cook

**Affiliations:** 1 Sir William Dunn School of Pathology, Medical Sciences Division, University of Oxford, Oxford, United Kingdom; 2 Laboratory for Systems Biology and Medicine, Research Center for Advanced Science and Technology, The University of Tokyo, Tokyo, Japan; National Cancer Institute, United States of America

## Abstract

It is widely assumed that active RNA polymerases track along their templates to produce a transcript. We test this using chromosome conformation capture and human genes switched on rapidly and synchronously by tumour necrosis factor alpha (TNFα); one is 221 kbp *SAMD4A*, which a polymerase takes more than 1 h to transcribe. Ten minutes after stimulation, the *SAMD4A* promoter comes together with other TNFα-responsive promoters. Subsequently, these contacts are lost as new downstream ones appear; contacts are invariably between sequences being transcribed. Super-resolution microscopy confirms that nascent transcripts (detected by RNA fluorescence in situ hybridization) co-localize at relevant times. Results are consistent with an alternative view of transcription: polymerases fixed in factories reel in their respective templates, so different parts of the templates transiently lie together.

## Abstract 2

Although it is widely assumed that active RNA polymerase tracks along its template, we find that DNA, not the polymerase, moves, suggesting that polymerase works by reeling in the template.

## Author Summary

We were all taught that an RNA polymerase becomes active by diffusing to a promoter, initiating transcription, and then tracking like a locomotive down the DNA template. We test this using tumour necrosis factor alpha (TNFα) to switch on transcription of two human genes which lie far apart on the genetic map and then measure how close the two are in 3D nuclear space. If what we were taught were true, there is no reason to expect the two genes to lie together. What we find—using two different techniques (cutting/ligating nearby sequences, and super-resolution microscopy)—is that the two genes are initially apart; then the parts of the genes being transcribed at a particular moment transiently come into close proximity. Our results are consistent with a model in which genes diffuse to a cluster of polymerases—a transcription factory—with transcripts being made as immobile polymerases reel in their respective templates. The DNA moves, not the polymerase.

## Introduction

It is widely assumed that an RNA polymerase transcribes by diffusing to a promoter, binding, and then tracking down the template as it makes its transcript [1]. Accumulating evidence, however, is consistent with an alternative: a promoter diffuses to a transcription factory where it binds to a transiently immobilized polymerase, which then reels in its template as it extrudes a transcript [2]–[6]. Here, we address the question: Are transcribing enzymes mobile or immobile?

Our strategy involves switching on transcription of two genes rapidly and synchronously using tumour necrosis factor alpha (TNFα). This cytokine orchestrates the inflammatory response in human umbilical vein endothelial cells (HUVECs) by signalling through nuclear factor kappa B (NF-κB) to activate a sub-set of genes [7]–[8]. *SAMD4A*—a 221 kbp-long gene that encodes a regulator of this pathway—is amongst the first few to respond. Microarray analysis reveals that a synchronous wave of transcription initiates within 15 min, before sweeping down the gene (at ∼3 kbp/min) to reach the terminus ∼70 min later (Figure S1); no transcripts from the non-coding strand are detected [9]. RNA FISH using intronic probes confirms that almost half the cells in the population respond; essentially no nascent RNA can be detected prior to stimulation, no transcription occurs from the antisense strand, and probes targeting successive introns only yield signal as the wave passes by [9].

**Figure 1:**
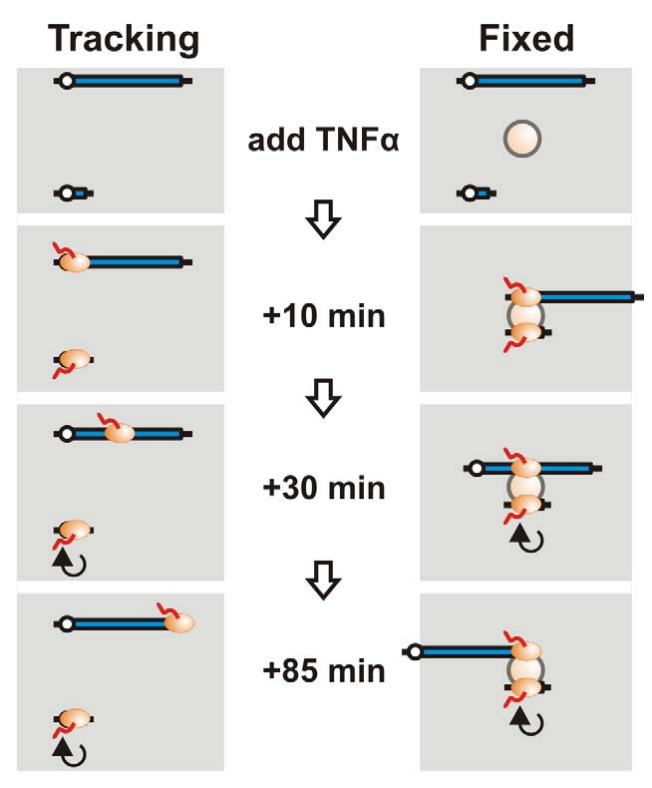
Distinguishing between tracking and fixed RNA polymerases. Before adding TNFα, both the long and short gene are not transcribed. Assuming they lie far apart on the same chromosome, they are unlikely to yield detectable 3C products. Ten min after adding TNFα, RNA polymerases (ovals) initiate on both genes. If active polymerases track, it remains unlikely that any part of the two genes will contact each other. However, if the two genes diffuse to one factory (sphere) and are then transcribed by transiently immobilized polymerases, the two promoters will lie close together. After 30 min, the pioneering polymerase on the short gene has terminated and been replaced by others that continuously transcribe it, while the pioneering polymerase on the long gene has only transcribed one-third of the gene. If polymerases track, the two genes are still unlikely to be together. But if polymerases are immobilized in a factory, the parts of the two genes currently being transcribed will lie together and yield a 3C product. After 85 min, the pioneering polymerase reaches the terminus of the long gene. If polymerases track, the two genes will still be apart; if immobilized, the terminus should now contact the short gene.

*TNFAIP2*—a short 11 kbp gene that lies ∼50 Mbp away from *SAMD4A* on chromosome 14—encodes another regulator. It is switched on as rapidly and then repeatedly transcribed over the next 90 min. We use it as an external reference point (or “anchor”) and analyze the contacts it makes with different parts of *SAMD4A* using chromosome conformation capture (3C)—a powerful tool for detecting proximity of two DNA sequences in 3D nuclear space [10]–[12]. If the conventional model for transcription applies, we would not expect the anchor to lie close to any part of *SAMD4A* either before or after adding TNFα, as it lies so far away on the chromosome (Figure 1, *left*). Even if polymerases on the two genes happened to lie together (for whatever reason), tracking of one down the long gene should increase the distance between transcribed parts of the two genes. But if both genes were transcribed by polymerases transiently immobilized in one factory, the short gene—which would repeatedly attach to (and detach from) the factory as it initiates (and terminates)—should always lie close to just the part of *SAMD4A* being transcribed at that particular moment (Figure 1, *right*). Thus, as the polymerase reels in *SAMD4A*, introns 1, 2, 3, etc. should successively be brought into the factory to lie transiently next to the anchor. Results using *TNFAIP2* (and other anchors) are impossible to reconcile with the widely held assumption that polymerases track; rather they are consistent with active polymerases being immobilized in factories.

## Results

### Some Interacting Partners of *SAMD4A* and Their Transcriptional Activation

As our strategy requires one gene to be used as an anchor, we applied 3C and a variant of “associated chromosome trap” (ACT) [13]–[14] to search for genes that interacted with *SAMD4A*. A number were found, and we chose four that were detected in independent experiments and which were relatively short (<60 kbp): *TNFAIP2*, *GCH1*, *PTRF*, and *SLC6A5* (Figure S2).

**Figure 2:**
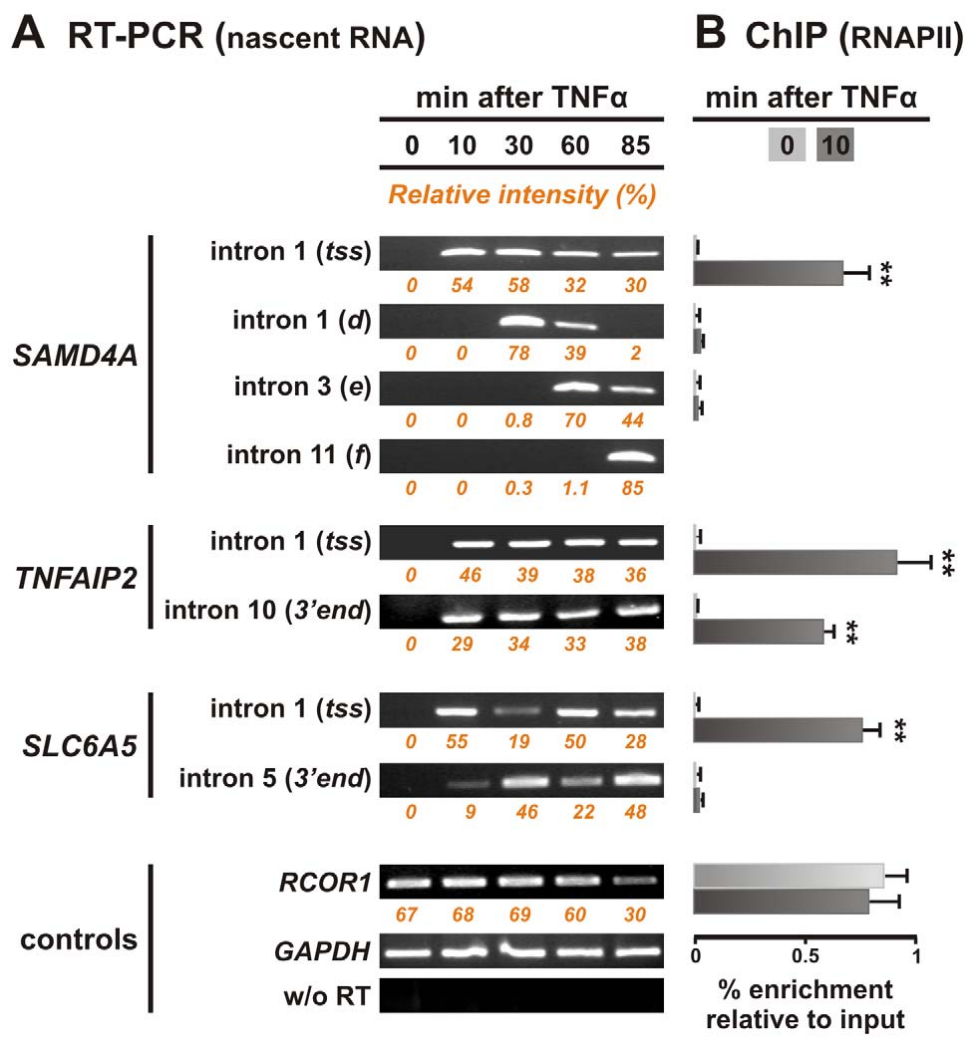
Polymerases initiate rapidly and synchronously on responding genes and elongate at expected rates. (A) Nascent RNA detected using reverse transcriptase PCR (RT-PCR). Total RNA was isolated from HUVECs 0–85 min after adding TNFα, treated with DNase, and intronic RNA detected. No nascent RNA copied from *SAMD4A*, *TNFAIP2*, or *SLC6A5* is detected at 0 min. For 221 kbp *SAMD4A*, nascent RNA appears at the *tss* after 10 min. As polymerases continue to initiate thereafter (albeit at declining rates), signal is seen at the *tss* between 15 and 85 min; however, many of these polymerases abort within ∼10 kbp of the *tss* (Figure S1; [9]). Nascent RNA from region *d* of intron 1 (34 kbp into the gene) is seen only after 30 min. Pioneering polymerases reach this region after 30 min and the slowest by 60 min; after 85 min all have passed by. Similarly, pioneering polymerases only reach introns 3 and 11 after 60 and 85 min, respectively. *TNFAIP2* is 11 kbp, and polymerases in the population can generate intronic RNA from both 5′ and 3′ ends within 10 min (it is then transcribed throughout the 85 min). *SLC6A5*—a 56 kbp gene—yields an intermediate pattern. No signal is again seen at 0 min, and pioneering polymerases generate maximal levels of intronic RNA at the *tss* after 10 min, and the 3′ end after 30 min; then, the cycle repeats between 60 and 85 min. Controls show levels of intronic RNA from two non-responsive, active genes (*GAPDH* and *RCOR1*), and that amplimers do not result from contaminating genomic DNA (using *GAPDH* probes, but replacing RT by *Taq* polymerase). Numbers under each panel (orange) correspond to the relative intensity of bands, corrected for background, and normalized to *GAPDH* levels. (B) Bound RNA polymerase II detected by chromatin immunoprecipitation (ChIP). Enrichments relative to input DNA (normalised to *GAPDH* levels) are shown 0 and 10 min after stimulation (light and dark grey bars, respectively). Almost no polymerase is bound to any part of *SAMD4A*, *TNFAIP2*, or *SLC6A5* at 0 min. The *tss* of *SAMD4A* is occupied by polymerases within 10 min of induction; however, levels further 3′ remain low as pioneering polymerases have not yet reached these regions. For *TNFAIP2*, some polymerases bind after 10 min, while others have reached the *3′ end* as the gene is so short. For *SLC6A5*, polymerases bind by 10 min to the *tss* but have not yet reached the 3′ end. *RCOR1* was analyzed as it was used as a control in RNA FISH experiments. Error bars show standard deviations from two independent experiments. **p<0.01, Student's *t* test compared to 0 min.

We initially verified that all five genes responded to TNFα by reverse-transcriptase PCR (RT-PCR). No intronic RNA (or only low levels in the case of *PTRF*) copied from the five genes was detected before induction, but higher levels were seen within 10 min of TNFα treatment (Figures 2A and S3F). Intronic RNA copied from further downstream in *SAMD4A* then appeared consistent with pioneering polymerases transcribing its 221 kbp at ∼3 kbp/min. Thus, RNA copied immediately downstream of the transcription start site (*tss*) appeared after 10 min, from ∼34 kbp into intron 1 after 30 min, from intron 3 after 60 min, and from the terminus after 85 min. In contrast, signal from each end of *TNFAIP2* is seen by 10 min. This 11 kbp gene is so short, and synchrony sufficiently poor, that some polymerases in the population are initiating as others are terminating (Figure 2A). *GCH1* and *SLC6A5*—both genes of ∼60 kbp—present intermediate patterns; pioneering polymerases reach termini after ∼30 min, before a second (reasonably synchronous) transcription cycle begins (Figures 2A and S3F). Such cycling has now been seen on various mammalian genes (e.g., [15]). Chromatin immunoprecipitation (ChIP) showed an enrichment of RNA polymerase II bound to the *tss* of all five genes within 10 min (Figures 2B and S3G). It also showed that NF-κB bound to promoters within 10 min (Figure S4), as might be expected [16]. RNA fluorescence in situ hybridization (FISH) also shows that intronic RNA copied from the relevant parts of the genes is present at the appropriate times (Figure S5). Therefore, results obtained with four independent methods (i.e., microarrays, RT-PCR, ChIP, RNA FISH) are in agreement and provide data on when polymerizing complexes are actively transcribing the sequences to be analyzed. These data are summarized in cartoons that accompany the results.

### Changing Contacts Between Two TNFα-Responsive Genes on Chromosome 14

**Figure 3 pbio-1000419-g003:**
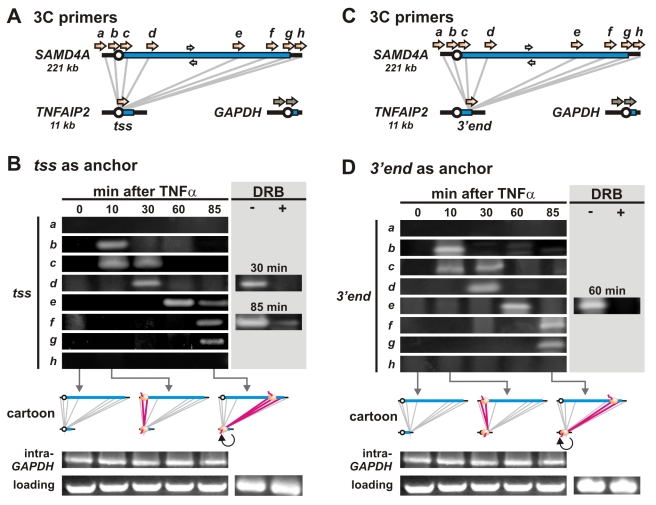
Contacts between two TNFα-responsive genes 50 Mbp apart on the same chromosome follow engaged polymerases. (A) Positions of 3C primers on *SAMD4A* and the *tss* of *TNFAIP2* (orange arrows) and *GAPDH* (grey arrows). Grey lines: 3C interactions monitored. White arrows: primers used for loading controls. (B) 3C. HUVECs were treated with TNFα for 0–85 min, 3C templates prepared using *Sac*I, and PCR conducted using equal weights of DNA and the primer pairs indicated; after gel electrophoresis and SYBR green staining, images of resulting gels are shown. The presence of a band reflects a high contact frequency between respective primer targets. Cartoons illustrate where polymerases are bound at different times and the interactions analyzed (grey lines); red lines indicate interactions yielding bands, and these always correlate with the presence of a polymerase on both contacting partners. In selected cases, DRB was added 20 min prior to harvesting cells (grey box); this reduces band intensity, indicating that contacts depend on transcription. *GAPDH* primers yield uniform levels of amplimers, as do loading controls. (C) Positions of 3C primers on *SAMD4A* and the *3′ end* of *TNFAIP2*. (D) Changing contacts between *SAMD4A* and the *3′ end* of *TNFAIP2*. The pattern is essentially the same as that in panel (B). Panels (B) and (D) share the same pair of loading and intra-*GAPDH* controls (excluding ± DRB), so the same image is shown in both panels.

Contacts between selected regions of *SAMD4A* and *TNFAIP2* were monitored by 3C, where the presence of a band after 34 PCR cycles reflects a high contact frequency (Figure 3). Essentially no contacts are seen between the *tss* of *TNFAIP2* (the anchor) and regions ∼25 kbp upstream or downstream of *SAMD4A* (*a*, *h*) at any time, or between the anchor and any region of *SAMD4A* (*b*–*g*) at 0 min—when no polymerases are engaged on either gene (Figure 3B, cartoon). By 10 min (when polymerases are first found on both genes; cartoon), contacts appear between the anchor and *SAMD4A* regions *b*, *c* (Figure 3B). Such contacts are soon lost, as new ones appear further 3′ on *SAMD4A*; they seem to steadily “slide” down the long gene. Thus, by 30 min, contacts are with regions *c* and *d*, by 60 min with region *e*, and by 85 min with regions *e*, *f*, and *g*. (The presence of more than one contact at certain times is consistent with imperfect synchrony amongst the ∼10^6^ cells assayed.) Treatment with DRB (5,6-dichloro-1-*β*-D-ribofuranosylbenzimidazole)—a reagent that inhibits transcription and releases polymerases from the template (Figure S6; [17]–[18])—reduces contacts (Figure 3B, *grey box*). Similar changing contacts were seen using (i) real-time PCR to quantify selected interactions (Figure S7), (ii) the *3′ end* of *TNFAIP2* as an anchor (Figure 3C, D; the gene is short enough for polymerases to be found at the same times on promoter and terminus in different cells in the population), and (iii) if *Hind*III replaced *Sac*I as the restriction enzyme used for 3C (Figure S8A, B). In every case, contacts are only seen at times when active polymerases are transcribing contacting sequences. Note that several genes lying within 50 Mbp on either side of *SAMD4A* do not interact with it (e.g., responsive *NFKBIA*, *SAV1*, *IRF9*, *GPR68*, and *PAPLN*; non-responsive *GMFB*, *YY1*, *HIF1A*, and *C14orf2*; and constitutive *RCOR1*; Figure S9A). As a whole, these results are inconsistent with the model involving tracking polymerases (Figure 1, *left*) but are simply explained if the two contacting templates are transiently tethered to polymerases fixed in one factory (Figure 1, *right*).

### Changing Contacts Between TNFα-Responsive Genes on Different Chromosomes

*PTRF* is a 21 kbp gene that lies on a different chromosome (i.e., 17) from *SAMD4A* (on 14). The pattern of interactions between the two is much the same as those seen between *SAMD4A* and *TNFAIP2* (Figure S3D, E), which is again consistent with the model involving fixed polymerases (Figure 1, *right*).

**Figure 4:**
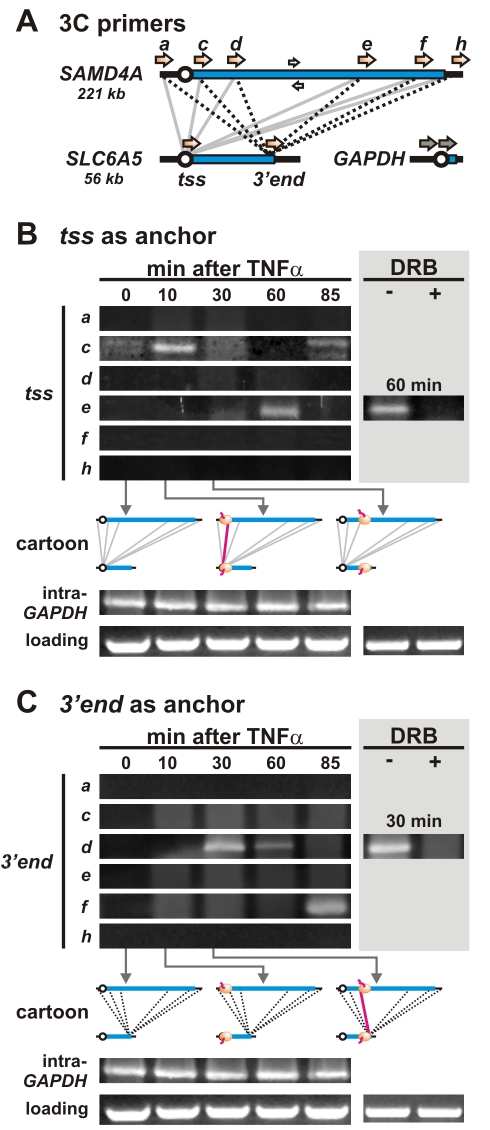
Contacts between two TNFα-responding genes on different chromosomes (14 and 11) follow engaged polymerases. (A) Positions of 3C primers and the interactions screened (grey and dotted black lines). (B) Contacts between the *tss* of *SLC6A5* (the anchor) and different parts of *SAMD4A*. Contacts/bands are only detected when polymerases are on both contacting partners. (C) Contacts between the *3′ end* of *SLC6A5* (the anchor) and different parts of *SAMD4A*. As in (B), two strong bands are seen, but they are in different positions. We suggest this is because it takes a polymerase 20–30 min to reach the *3′ end* of *SLC6A5* now used as an anchor; then, contacts/bands are again only detected when polymerases are on both contacting partners. Panels (B) and (C) share the same pair of loading and intra-*GAPDH* controls (excluding ± DRB), so the same image is shown in both panels.

A more complex pattern of changing contacts is seen between *SAMD4A* and a 60 kbp gene on chromosome 11, *SLC6A5* (Figure 4); this pattern suggests that polymerases must be present on both contacting sequences. Thus, as before, no contacts are seen between the *tss* of *SLC6A5* (the anchor) and regions upstream or downstream of *SAMD4A* (*a*, *h*) at any time, or between the anchor and any region of *SAMD4A* at 0 min—when no polymerases are engaged on either gene (Figure 4B, cartoon). Again as before, contacts appear between the anchor and *SAMD4A* region *c* (which includes the *tss* and the beginning of intron 1) after 10 min (Figure 4B), when polymerases are first found on both. But after 30 min (when contacts with region *d* were seen in Figure 3B), essentially no contacts are found (Figure 4B). This is consistent with pioneering polymerases leaving the *tss* of the anchor so that they are now transcribing the *3′ end* of this ∼60 kbp gene, as data in Figure 2 indicate. By 60 min (when a second polymerase is just initiating on the *tss* of *SLC6A5*; Figure 2), we see a strong (second) contact with the region on *SAMD4A* that its pioneering polymerase is now transcribing (i.e., *e* in Figure 4B). This interaction is DRB-sensitive (Figure 4B, *grey box*), and so depends on continuing transcription. No prominent interactions are seen at 85 min (Figure 4B) even though we know *SAMD4A* is still being transcribed. Moreover, the contact seen with region *f* in Figure 3B is missing, presumably because the second polymerase on *SLC6A5* has left the *tss* used as the anchor and is now transcribing the 3′ end (Figure 2). An almost identical pattern with analogous missing contacts is seen if *Hind*III replaces *Sac*I during preparation of the 3C template (Figure S8A, C).

If the above explanation is correct, with contacts only being seen if active polymerases are present on both contacting partners, then use of the 3′ end of *SLC6A5* as an anchor should change the pattern as follows. The two bands seen in Figure 4B should disappear (as polymerases at the relevant times are on the *tss* and not the *3′ end* now used as the anchor), while the two “missing” bands should reappear (as polymerases have now reached the *3′ end*); they do. For example, comparison of Figure 4B and C shows that the first missing band/contact (with *d* at 30 min in Figure 3B) reappears in Figure 4C, as does the second (with *f* at 85 min). Bands/contacts are also sensitive to DRB (Figures 4B,C, *grey boxes*).

This interpretation is reinforced by an analysis involving 5′ and 3′ anchors on another gene (of similar length as *SLC6A5*) that lie on the same chromosome as *SAMD4A*. Thus, *GCH1* is ∼0.8 Mbp away from *SAMD4A* and responds as rapidly to TNFα (Figure S3F, G). When its 5′ and 3′ ends are used as anchors, a complex set of changing contacts (and missing bands) is again seen (Figure S3A–C).

We also confirmed that the *tss* of *GCH1* lay next to the *tss* of *TNFAIP2* at 10 min but not at 0 min (Figure S9A). This is consistent with responding promoters coming together to the same factory when active. As all other contacts analyzed involve *SAMD4A*, these results also indicate that such reorganization is not peculiar to one long gene.

### Nascent RNAs Also Colocalize at the Appropriate Times

**Figure 5:**
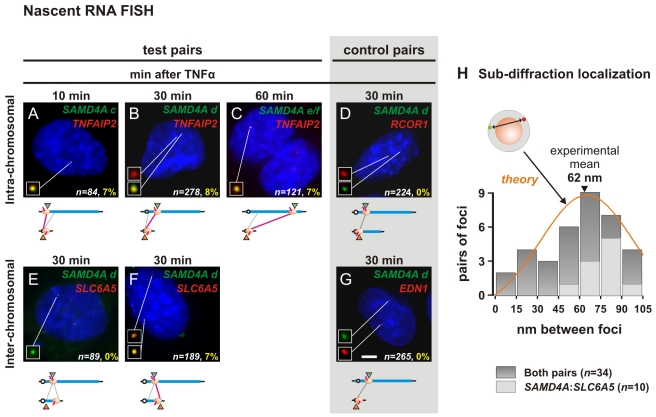
Colocalization of intronic RNA demonstrated by RNA FISH. HUVECs were treated with TNFα for 10, 30, or 60 min, and nascent RNAs copied from test and control pairs of genes detected by RNA FISH. (A–C) Colocalization of nascent RNAs encoded by genes on the same chromosome. The two probes target RNA copied from intron 2 of *TNFAIP2* (red) and intronic region *c*, or *d*, or *e/f* of *SAMD4A* (green); representative images of DAPI-stained nuclei are shown (insets provide magnifications of foci indicated). Red and green foci mark (non-colocalizing) nascent transcripts copied from one (or both) allele, and yellow foci colocalizing ones; *n* gives the number of alleles active in all cells analyzed that have ≥1 green focus plus ≥1 red focus. Numbers in yellow give the fraction of colocalizing red and green foci (where >75% pixels in one focus share red and green signal) expressed as a percentage of *n*; values were significantly different from those seen in (D) with a control gene (*p*<10^−3^, Fischer's exact test, one-tailed). The cartoon illustrates the targets of red and green probes (triangles), and the positions of polymerases; red lines between targets indicate that yellow foci were detected (grey lines: no yellow foci detected—see Figure S5C). (D) Yellow foci were never seen with probes targeting intronic RNA copied from *SAMD4A* and a (non-responsive) control gene (*RCOR1*) that lies between *SAMD4A* and *TNFAIP2*. (E–F) Colocalization of nascent RNAs encoded by genes on different chromosomes. The two probes target RNA copied from intronic region *d* of *SAMD4A* (green) and either intron 1 or 10 of *SLC6A5* (red); only the latter yields yellow foci (the number of yellow foci was significantly different from that seen in (G) with a control gene; *p*<10^−3^, Fischer's exact test, one-tailed). (G) Yellow foci were never seen with probes targeting intronic RNA copied from *SAMD4A* and a (non-responsive) control gene (*EDN1*) that lies on a different chromosome from *SAMD4A*. Bar: 5 µm. (H) Sub-diffraction localization of peaks of red and green signal within yellow foci. Gaussian curves were fitted to the intensities of the red and green signals, and distances between peaks determined with a precision of 15 nm (see Methods for details). Dark grey bars illustrate distances obtained from 34 yellow foci seen in images like those in (B) and (F); the mean distance is 62 nm. Light grey bars illustrate similar distances obtained from 10 yellow foci like that in (F). The model shows a red and green point randomly distributed in a 35 nm shell (grey) around an 87 nm diameter factory (orange sphere); simulations using this model yield the distribution indicated (orange line).

If responding regions only lie together when transcribed, their nascent transcripts should also only be together at the appropriate times. To test this we used RNA FISH with pairs of probes each able to detect an intron within a single nascent transcript copied RNA transcript at its transcription site; colocalization of nascent transcripts copied from the two different genes then yields a yellow focus [9],[19]. Yellow foci were given by the *TNFAIP2* probe (red) and *SAMD4A* probes *c*, *d*, and *e/f* (green) at 10, 30, and 60 min post-induction (Figure 5A–C). No such colocalization was seen at other times (Figure S5), when relevant regions were not being transcribed. As a control, we analyzed nascent transcripts copied from a non-responsive (constitutively-active) gene—*RCOR1*—that lies between *SAMD4A* and *TNFAIP2* (Figure S9A); no yellow foci were detected (Figure 5D). Just as 3C showed the templates lie together (Figure 2), RNA FISH confirms their transcripts also colocalize.

We also investigated inter-chromosomal contacts 30 min post-induction, using probes targeting (green) *SAMD4A* region *d* and (red) *SLC6A5* intron 1 (close to the *tss*) or intron 10 (close to the *3′ end*). When no 3C contacts between *SAMD4A* region *d* and the *tss* of *SLC6A5* were seen (Figure 4B), no yellow foci were detected (Figure 5E; Figure S5C). But the “missing” 3C band was seen at 30 min using the 3′ terminus as anchor (Figure 4C), and then yellow foci are seen (Figure 5F). As a control, we analyzed nascent transcripts copied from another non-responsive (constitutively-active) gene—*EDN1*—that lies on a different chromosome; again, no yellow foci were seen (Figure 5G).

### Super-Resolution Localization of Nascent Transcripts

Electron microscopy reveals that nascent nucleoplasmic transcripts typically lie on the surface of ∼87 nm (protein-rich) factories [20]. To see if colocalizing transcripts encoded by the *SAMD4A d*:*TNFAIP2* and *SAMD4A d*:*SLC6A5* pairs lie this close together, we used a new approach that allows resolution beyond the diffraction limit of the light microscope [21]–[23]. We assume the red and green signals that yield a yellow focus (e.g., Figure S5B) mark two sub-diffraction spots, fit Gaussian curves to their intensities, and measure the distance (with 15 nm precision) between peaks [23]; the distance between the two transcripts ranges from 7 to 102 nm, with a mean separation of 62 nm (Figure 5H). This distribution is much like that seen when a pair of red and green points are repeatedly and randomly distributed in a 35 nm shell surrounding an 87 nm diameter sphere (Figure 5H, orange line). [Subdiffraction-sized red/green fluorescent beads of 110 nm serve as a truly co-localizing control (Figure S5B, left); then, the distance between their red and green peaks is within the uncertainty of our measurements (*n* = 8; not shown).] These results are consistent with nascent transcripts copied from the two different genes lying on the surface of the same transcription factory.

## Discussion

We tested the two models illustrated in Figure 1 to address one fundamental assumption of modern molecular biology, namely that a transcribing polymerase tracks along its template as it makes its transcript. *SAMD4A* has a unique set of properties that make it particularly useful for this analysis; it can be switched on rapidly and synchronously by TNFα (with approximately half the cells in the population responding), its length provides sufficient temporal and spatial resolution (it takes ∼70 min to transcribe, and contains many restriction sites that facilitate the use of 3C to discriminate between contacts produced by different parts of the gene), and neither its sense or anti-sense strands encode other transcription units that might complicate analysis. 3C reveals that just the parts of *SAMD4A* being transcribed at a particular moment lie close to just the parts of three other genes being transcribed at that moment (Figures 3, 4, S3, and S8). These inter-genic contacts occur infrequently, as expected [24]–[26]. RNA FISH confirmed that the relevant nascent RNAs lie together at the appropriate times (Figures 5 and S5), while “super-resolution” microscopy (allowing measurements below the diffraction limit) showed that the distance between the two transcripts is consistent with them lying within 35 nm of the surface of an 87 nm sphere (Figure 5H). Such results are difficult—if not impossible—to explain if polymerases track. Rather, they are consistent with an alternative where two responding genes diffuse to an 87 nm factory to be transcribed by immobilized enzymes. Then, as the two genes are reeled in, only parts being transcribed at a given moment will lie transiently together [5].

These results beg many questions. For example, we were able to detect interacting sequences at a reasonable frequency simply by assuming the existence of factories dedicated to transcribing genes that respond rapidly to TNFα (Figures S2 and S9). If such specialized factories exist [27],[28], how many might there be in a nucleus, and how many are accessible to a gene like *SAMD4A*? Fortunately, these questions will soon be answered, as techniques for analyzing all contacts made by any gene in a nucleus have been developed [29]. We also note that our results are consistent with others obtained from a recent genome-wide study; after stimulating human cells with estrogen and mapping contacts made by bound estrogen receptor-α (using ChIP, 3C, and “deep” sequencing), contacting partners were often associated with bound RNA polymerase II [30].

## Materials and Methods

A detailed description of the experimental procedures is given in Text S1.

### Cell Culture

HUVECs from pooled donors (Lonza) were grown to 80%–90% confluency in Endothelial Basal Medium 2-MV with supplements (EBM; Lonza), starved (18 h) in EBM+0.5% FBS, and treated with TNFα (10 ng/ml; Peprotech) for up to 85 min. In some cases, 50 µM 5,6-dichloro-1-*β*-D-ribofuranosylbenzimidazole (DRB; Sigma-Aldrich) was added 20 min before harvesting cells.

### 3C

3C was performed as described [10]. In brief, 10^7^ cells were fixed (10 min; room temperature) in 1% paraformaldehyde (Electron Microscopy Sciences), “Dounce”-homogenized, and membranes lyzed (30 min; 4°C) using 0.2% Igepal (Sigma-Aldrich). Nuclei were pelleted and resuspended in the appropriate restriction buffer, incubated (16 h; 37°C) with *Sac*I or *Hind*III (800 units/10^6^ cells; New England Biolabs), diluted to 8 ml in ligation buffer, T4 DNA ligase added (4,000 units/10^6^ cells; New England Biolabs), and incubated (48 h at 4°C, then 20 min at room temperature). After reversing cross-links (16 h; 65°C), DNA was purified by phenol extraction and ethanol precipitation, cut with *Bgl*II to reduce fragment length, and repurified. 71%–78% restriction sites in the template were cut by *Sac*I or *Hind*III (determined as in [31]). PCR conditions were adjusted so that reactions were within the linear range of amplification (i.e., ∼175 ng template/reaction; 1.75 mM MgCl_2_, 1% dimethylsulphoxide, 10 pmoles of each primer, and GoTaq polymerase (Promega); 95°C for 2 min, then 34 cycles at 95°C for 55 s, 59°C for 45 s, and 72°C for 20 s, followed by one cycle at 72°C for 2 min); amplimers were resolved on 2.5% agarose gels, stained with SYBR Green (Invitrogen), and scanned using an FLA-5000 scanner (Fuji). Identities of all 3C products were confirmed by DNA sequencing (Geneservices, Oxford), except for those in Figure S8 (where identities were confirmed by restriction digestion). Amplification efficiencies were examined using a control template generated by *Sac*I or *Hind*III digestion of BAC clones covering *GAPDH* on HSA12 (RP5-940J5; ImaGenes), *SAMD4A*, *GCH1* (RP11-170J16, CTC-775N1, CTD-2586I5, CTD-2378G4; CHORI, Invitrogen), and *TNFAIP2* (CTD-2594N9; Invitrogen) on HSA14, *SLC6A5* on HSA11 (RP11-120F6; CHORI), and *PTRF* on HSA17 (RP11-194N12; CHORI) followed by ligation. This synthetic template was spiked (to reach 175 ng/µl) with HUVEC DNA cut with the relevant restriction enzyme and ligated. Other control templates included non-digested/ligated DNA and digested/non-ligated DNA (both from 10^6^ cells). Results shown were reproduced using at least two independently obtained templates.

## Supporting Information

Figure S1 **TNFα induces a wave of transcription to sweep along** ***SAMD4A*** **.** HUVECs were treated with TNFα, samples collected every 7.5 min for 3 h, total RNA purified and hybridized to a tiling microarray bearing 25-mers complementary to *SAMD4A* (modified from [9]). On the gene map (top) positions of introns, exons, and targets of 3C primers *a–h* are indicated. Position *a* corresponds to 25 kbp 5′ before the transcription start site (*tss*), *b* to the promoter, *c* to the beginning of intron 1, *d* to 34 kbp into intron 1, *e* to intron 3, *f* to intron 11, *g* to the 3′ untranslated region (*utr*), and *h* to 25 kbp after the poly(A) site. The vertical axis gives intensity of signal of intronic and exonic probes (red and yellow vertical needles, respectively); genomic location (bottom) and time after stimulation (top to bottom) are shown. No transcripts copied from either sense or anti-sense strands are detected at 7.5 min [9]. A wave of signal initiates at the 5′ end within 15 min (start), and then travels down the gene to terminate after 75–90 min (end). Co-transcriptional splicing and premature termination conspire to generate this wave (e.g., as the wave reaches the middle of intron 2 after 60–75 min, little signal is seen in intron 1). Note also that probes covering the first thousands of nucleotides from the *tss* yield signal between 15–180 min, and polymerases only seem to escape downstream in a limited interval (i.e., after 15–30 min) to initiate a first, fairly synchronous wave. This points to a checkpoint regulating escape; it seems to act on a second polymerase once it senses there is already a first on the gene (despite being perhaps 100 kbp downstream). This figure is reproduced from [9].
(1.27 MB TIF)

Figure S2 **Changing contacts detected using “circular ACT” (associated chromosome trap).** To detect intra-/inter-chromosomal contacts made by *SAMD4A* regions *c* and *d* at 0, 10, and 30 min after adding TNFα, we performed circular ACT [13],[14]. 3C templates were prepared using either *Sac*I or *Hind*III and then *Csp*6I, nested inverse PCR conducted (using primers targeting *SAMD4A* regions *c* or *d*), products cloned and sequenced, and segments contacting *SAMD4A* mapped. Genic contacts with gene name, region of gene, chromosomal location, and the number of times (hits) that particular sequence was seen compared to the total number of sequences analyzed (includes self-ligation products and contacts with non-coding regions that are not shown) are listed. Results support the idea that, at 0 min, *SAMD4A* makes few contacts. After 10 min, region *c* contacts many more genes, including partners (highlighted) we study (*TNFAIP2*, *GCH1*, *SLC6A5*, *PTRF*); no such contacts are seen with region *d* (the wave of transcription has not yet reached this region). After 30 min, region *d* now contacts *TNFAIP2* and *SLC6A5* (in accord with 3C data in Figures 3 and 4; note a contact between *SAMD4A* and the *tss* of *SLC6A5* is detected at 10 min, and one with the 3′ end of *SLC6A5* at 30 min). In a population of cells, a gene contacts other genomic regions with varying frequencies [26],[27], and circular ACT detects those occurring the most often (to give repeated “hits” in independent experiments) against an inevitable background [13],[14]. As in independent experiments we detect contacts between *SAMD4A* and *TNFAIP2*, *SLC6A5*, *PTRF1* (shown here), and *GCH1* (one contact shown here, plus one additional one seen after 60 min; not shown), it is likely that all these interactions are major ones—although not necessarily the strongest ones.
(0.41 MB TIF)

Figure S3 **Contacts between** ***SAMD4A*** **and** ***GCH1*** **(or** ***PTRF*** **) follow engaged polymerases.** General details are as in Figure 3A. (A) Positions of 3C primers targeting *SAMD4A* and *GCH1*, which lie ∼0.8 Mbp apart on chromosome 14. (B, C) Contacts between *SAMD4A* and the 5′ and 3′ ends of *GCH1*. The interaction pattern is similar to that seen with *SAMD4A* and *SLC6A5* (which is of comparable length to *GCH1*; Figure 4). Panel (B) shares with (C) the same intra-*GAPDH* and loading controls (excluding ± DRB). (D) Positions of 3C primers targeting *SAMD4A* and *PTRF*. (E) Contacts between *SAMD4A* and the *tss* of *PTRF* (on chromosome 17). The interaction pattern is similar to that seen between *SAMD4A* and *TNFAIP2* (Figure 3). (F) Nascent RNA detected by RT-PCR in total RNA isolated from HUVECs 0–85 min after adding TNFα. For *GCH1* at 0 min, no signal is seen. After 10 min, maximal levels of RNA are seen at the *tss* (intron 1); after 30 min, they are seen at the *3′ end* (intron 5). This cycle repeats between 60 and 85 min. *PTRF* is expressed prior to TNFα induction, but levels of intronic RNA increase after stimulation. Controls show that levels of *GAPDH* intronic RNA remain unchanged and that amplimers do not result from contaminating genomic DNA (w/o RT). (G) Levels of bound RNA polymerase II (detected by ChIP using anti-phospho-Ser5 in the C-terminal domain of the largest subunit) 0–10 min after stimulation (light and dark grey bars, respectively). Levels of enrichment are expressed relative to those of the input; values for different amplicons are normalised relative to those seen with *GAPDH*. Error bars show standard deviations from two independent experiments. *p<0.05, **p<0.01, Student's *t* test compared to 0 min.
(1.11 MB TIF)

Figure S4 **NF-κB binds to promoters of TNFα-responding genes within 10 min.** HUVECs were treated with TNFα, and binding of NF-κB (p65 subunit) assessed by ChIP using chromatin obtained 0–10 min (light and dark grey bars, respectively) post-induction. Putative NF-κB binding sites (5′-GGGRNNYCC-3′; red boxes) in the 5′ proximal regions of five genes are indicated; the *GMFB* promoter region (white box) contains no such sites and serves as a negative control. Bars over each targeted region show the percentage enrichment relative to input DNA. Error bars show standard deviations from three independent experiments. *p<0.05, **p<0.01, Student's *t* test compared to 0 min.
(0.19 MB TIF)

Figure S5 **Summary of RNA FISH results.** (A) Positions of RNA FISH probes that target introns within *SAMD4A* (green triangles), *TNFAIP2*, and *SLC6A5* (red triangles). (B) Criteria used to assess overlap of red and green foci. The image on the left provides a colocalizing control: a 110 nm bead that fluoresces in both red and green channels to give yellow in this merged image. The images in the middle and on the right are of foci collected as in Figure 5F using probes targeting *SAMD4A* region *d* (green) and *SLC6A5* intron 5 (red) 30 min after induction. A focus is defined as >4 contiguous (90 nm) pixels that contain signal above a threshold (defined as the average intensity of at least 50 pixels in a line-scan across the focus); typically, foci were 12±4 pixels in size and were classified as red or green (no signal of the other colour above threshold in >75% pixels) or yellow (signal above threshold of both colours in ≥75% pixels). The middle image is therefore scored as one red and one green focus even though the two partially overlap; such partially overlapping foci were rare (constituting <3% of all foci). The image on the right is scored as a yellow focus (as >75% pixels in the focus contain both green and red signals above the threshold). Bar: 200 nm. (C) Summary of RNA FISH results. HUVECs were treated with TNFα for 10–60 min, RNA FISH performed with probe pairs detecting nascent RNA copied from the regions indicated, and numbers of cells containing red, green, and yellow foci determined (from images like those in Figure 5A–G). In each case, one probe (green) targets RNA copied from regions *c*, *d*, or *e/f* of *SAMD4A*, while a second (red) targets intronic RNA from either a control gene that yields no 3C product with *SAMD4A* (i.e., *RCOR1*, *EDN1*) or a test gene (i.e., *TNFAIP2*, *SLC6A5*) that does. Values represent numbers of cells (*n*) with the patterns indicated (percentages in brackets); numbers of yellow foci are highlighted. A probe targeting the anti-sense strand of *SAMD4A* region *d*, and pretreatment of cells with RNase A yields no signal (not shown). Before induction, probes targeting *TNFAIP2*, *SLC6A5* introns 1 and 10, and *SAMD4A* regions *d* and *e/f* yield no foci; *SAMD4A* probe *c* yields foci in <3% cells. Results confirm polymerase positionings and 3C results (Figures 2–4). For example, at 10 min essentially no cells with green foci marking *SAMD4A* region *d* are seen, as this region is not yet transcribed; however, a significant number are seen after 30 min when it is. Similarly, many red foci marking *SLC6A5* intron 10 are seen after 30 min, but not after 10 min (and the opposite applies to foci marking *SLC6A5* intron 1). However, red foci are seen at both times with the short gene, *TNFAIP2*. No yellow foci were seen at any time with probe pairs targeting transcripts copied from region *d* and a control gene (*RCOR1*, *EDN1*). In contrast, probe pairs targeting *SAMD4A* and the test genes did yield yellow foci at times when polymerases were transcribing the appropriate regions (cartoons). These differences in the numbers of yellow foci are small but statistically significant. Consider, for example, the *SAMD4A d*:*RCOR1* pair at 30 min. In cells with ≥1 green plus ≥1 red focus (values within the orange box), there were 224 active alleles, but none overlapped to give a yellow focus. But with the *SAMD4A d*:*TNFAIP2* pair, 22 out of the 278 active alleles (i.e., 8%) overlapped to give a yellow focus. This difference is significant (*p* = 3.3×10^−6^; Fisher's exact test, one-tailed). Similarly, for the *SAMD4A d*:*EDN1* pair at 30 min, none of the 265 active alleles overlapped. However, in the *SAMD4A d*:*SLC6A5*-intron-10 pair, 14 of the 189 active alleles (i.e., 7%) overlapped. The difference between the two pairs was again significant (*p* = 6×10^−6^; Fisher's exact test, one-tailed).
(1.58 MB TIF)

Figure S6 **Binding of phosphorylated forms of RNA polymerase II along** ***SAMD4A*** **.** ChIP was performed using antisera predominantly recognizing the largest subunit of RNA polymerase II phosphorylated at serine 5 (H14; red curves) or serine 2 (3E10; blue curve) in the heptad repeats of the C-terminal domain [18]. The cartoon below indicates probe positions. Chromatin was isolated from HUVECs 0 or 30 min after induction; in some cases DRB was added 20 min before harvesting. For the first two panels, ChIP-chip results (blue) adapted from [9] are included. At 0 min, little signal is seen along the gene; at 30 min, significant amounts of the polymerase are bound on the first third of the gene. Upon DRB treatment (bottom panel), phospho-serine 2 signal returns to background levels, whereas phospho-serine 5 signal accumulates around the *tss*, as might be expected [17],[18]. Experiments were performed on two independently prepared templates; error bars show standard deviations (*p<0.05, **p<0.01, Student's *t* test compared to 30 min).
(2.44 MB TIF)

Figure S7 **Selected 3C interactions assessed by quantitative real-time PCR.** HUVECs were treated with TNFα for 0–30 min, 3C templates prepared using *Sac*I, and qPCR conducted using equal weights of DNA and primers targeting indicated regions; amounts of 3C products detected were normalized relative to intra-*GAPDH* 3C amplimers (as in [27]). In some cases DRB was added 20 min before harvesting cells. Cartoons illustrate where polymerases are bound at different times and the interactions analyzed (grey lines); red lines indicate interactions detected, and these correlate with the presence of a polymerase on both partners. Values are averages (± standard deviation) from three independent experiments. (A) Interactions between *SAMD4A* fragment *c* and four TNFα-responsive genes. Strong interactions are seen with three genes (but not *NFKBIA*). (B) Interactions between *SAMD4A* fragments *b–d* and *TNFAIP2*. Strong interactions are seen at appropriate times, confirming results in Figure 3. As DRB inhibits productive elongation (see Figure S6), interactions around the promoter and *tss* are still detected.
(0.32 MB TIF)

Figure S8 **Using** ***Hind*** **III to prepare 3C templates yields interactions like those seen with** ***Sac*** **I.** Details are as for Figures 3 and 4; essentially the same changing patterns are detected. (A) Positions of 3C primers. (B) Interactions between *SAMD4A* and *TNFAIP2*. (C) Interactions between *SAMD4A* and the *tss* of *SLC6A5*.
(0.63 MB TIF)

Figure S9 **Some 3C controls. (A) Specificity of inter-genic interactions.** Genes screened (TNFα-responsive, non-responsive, and constitutive) are indicated on the map of part of human chromosome 14 (from genome reference assembly 37). HUVECs were treated with TNFα for 0–10 min, 3C templates prepared using *Sac*I, and PCR conducted using primers targeting the *tss* of each gene. *SAMD4A* contacts the TNFα-responsive gene *TNFAIP2* (grey arrow) which lies ∼50 Mbp downstream, but not another responding gene—*NFKBIA*—lying ∼20 Mbp upstream, nor two non-responsive genes—*GMFB*, *RCOR1*—lying ∼0.1 and ∼40 Mbp downstream. [Additional responding non-interactors included *SAV1*, *IRF1*, *GPR68*, and *PAPLN*; additional non-responding non-interactors included *YY1*, *HIF1A*, *C14orf2* (not shown).] Responsive genes *GCH1* and *TNFAIP2* also contact one another (grey arrow). 3C products obtained from two parts of *GAPDH* yield uniform levels of amplimers, as do loading controls. (B) Controls for amplification efficiencies of primers. Amplification efficiencies were assessed using a control template generated by digestion of BAC clones with *Sac*I followed by ligation. As in (A), PCR was conducted using equal weights of these templates and primers targeting regions indicated. Different primer pairs yield comparable amounts of amplimers. (C) 3C conducted using serial 2-fold dilutions of template to assess the range of linear amplification. In the examples shown, 3C templates are derived from HUVECs treated with TNFα for 10 min; 1× dilution represents 200 ng of template per 25 µl reaction volume. 3C reactions shown in all other figures were adjusted accordingly.
(0.97 MB TIF)

Text S1 **Supplementary information.** Detailed Materials and Methods.
(0.05 MB DOC)

## Abbreviations

3C: chromosome conformation capture
ACT: associated chromosome trap
ChIP: chromatin immunoprecipitation
FISH: fluorescence in situ hybridization
HUVECs: human umbilical vein endothelial cells
NF-κB: nuclear factor kappa B
RT-PCR: reverse-transcriptase PCR
TNFα tumour necrosis factor alpha
*tss*: transcription start site

## References

[1] Alberts B, Johnson A, Lewis J, Raff M, Roberts K, Walter P (2002). Molecular biology of the cell 4th edition.

[2] Jackson D. A, McCready S. J, Cook P. R (1981). RNA is synthesised at the nuclear cage.. Nature.

[3] Cook P. R (1999). The organization of replication and transcription.. Science.

[4] Sexton T, Umlauf D, Kurukuti S, Fraser P (2007). The role of transcription factories in large-scale structure and dynamics of interphase chromatin.. Semin Cell Dev Biol.

[5] Cook P. R (2009). A model for all genomes; the role of transcription factories.. J Mol Biol.

[6] Sutherland H, Bickmore W. A (2009). Transcription factories: gene expression in unions?. Nat Rev Genet.

[7] Hoffmann A, Baltimore D (2006). Circuitry of nuclear factor kappaB signaling.. Immunological Reviews.

[8] Bradley J. R (2008). TNF-mediated inflammatory disease.. J Pathol.

[9] Wada Y, Ohta Y, Xu M, Tsutsumi S, Minami T, Inoue K, Komura D, Kitakami J, Oshida N, Papantonis A (2009). Visualizing a wave of transcription as it sweeps along activated human genes.. Proc Natl Acad Sci U S A.

[10] Miele A, Gheldof N, Tabuchi T. M, Dostie J, Dekker J (2006). Mapping chromatin interactions by chromosome conformation capture.. Curr Protoc Mol Biol.

[11] Simonis M, de Laat W (2008). FISH-eyed and genome-wide views on the spatial organisation of gene expression.. Biochim Biophys Acta.

[12] Göndör A, Ohlsson R (2009). Chromosome crosstalk in three dimensions.. Nature.

[13] Ling J. Q, Li T, Hu J. F, Vu T. H, Chen H. L, Qiu X. W, Cherry A. M, Hoffman A. R (2006). CTCF mediates interchromosomal colocalization between Igf2/H19 and Wsb1/Nf1.. Science.

[14] Würtele H, Chartrand P (2006). Genome-wide scanning of HoxB1-associated loci in mouse ES cells using an open-ended chromosome conformation capture methodology.. Genome Res.

[15] Degenhardt T, Rybakova K. N, Tomaszewska A, Moné M. J, Westerhoff H. V, Bruggeman F. J, Carlberg C (2009). Population-level transcription cycles derive from stochastic timing of single-cell transcription.. Cell.

[16] Ashall L, Horton C. A, Nelson D. E, Paszek P, Harper C. V, Sillitoe K, Ryan S, Spiller D. G, Unitt J. F, Broomhead D. S (2009). Pulsatile stimulation determines timing and specificity of NF-kappaB-dependent transcription.. Science.

[17] Kimura H, Sugaya K, Cook P. R (2002). The transcription cycle of RNA polymerase II in living cells.. J Cell Biol.

[18] Chapman R. D, Heidemann M, Albert T. K, Mailhammer R, Flatley A, Meisterernst M, Kremmer E, Eick D (2007). Transcribing RNA polymerase II is phosphorylated at CTD residue serine-7.. Science.

[19] Femino A. M, Fay F. S, Fogarty K, Singer R. H (1998). Visualization of single RNA transcripts in situ.. Science.

[20] Eskiw C. H, Rapp A, Carter D. R, Cook P. R (2007). RNA polymerase II activity is located on the surface of protein-rich transcription factories.. J Cell Sci.

[21] Thompson R. E, Larson D. R, Webb W. W (2002). Precise nanometer localization analysis for individual fluorescent probes.. Biophysical J.

[22] Yildiz A, Forkey J. N, McKinney S. A, Ha T, Goldman Y. E, Selvin P. R (2003). Myosin V walks hand-over-hand: single fluorophore imaging with 1.5-nm localization.. Science.

[23] Larkin J. D, Publicover N. G, Sutko J. L (2010). Photon event distribution sampling: an image formation technique for scanning microscopes permits tracking of sub-diffraction particles with high spatial and temporal resolution.. J Microscopy.

[24] Osborne C. S, Chakalova L, Brown K. E, Carter D, Horton A, Debrand E, Goyenechea B, Mitchell J. A, Lopes S, Reik W, Fraser P (2004). Active genes dynamically colocalize to shared sites of ongoing transcription.. Nat Genet.

[25] Zhao Z, Tavoosidana G, Sjölinder M, Göndör A, Mariano P, Wang S, Kanduri C, Lezcano M, Sandhu K. S, Singh U (2006). Circular chromosome conformation capture (4C) uncovers extensive networks of epigenetically regulated intra- and interchromosomal interactions.. Nat Genet.

[26] Dhar S. S, Ongwijitwat S, Wong-Riley M. T (2009). Chromosome conformation capture of all 13 genomic loci in the transcriptional regulation of the multisubunit bigenomic cytochrome C oxidase in neurons.. J Biol Chem.

[27] Pombo A, Jackson D. A, Hollinshead M, Wang Z, Roeder R. G, Cook P. R (1999). Regional specialization in human nuclei: visualization of discrete sites of transcription by RNA polymerase III.. EMBO J.

[28] Xu M, Cook P. R (2008). Similar active genes cluster in specialized transcription factories.. J Cell Biol.

[29] Lieberman-Aiden E, van Berkum N. L, Williams L, Imakaev M, Ragoczy T, Telling A, Amit I, Lajoie B. R, Sabo P. J, Dorschner M. O (2009). Comprehensive mapping of long-range interactions reveals folding principles of the human genome.. Science.

[30] Fullwood M. J, Liu M. H, Pan Y. F, Liu J, Xu H, Mohamed Y. B, Orlov Y. L, Velkov S, Ho A, Mei P. H, Chew E. G (2009). An oestrogen-receptor-alpha-bound human chromatin interactome.. Nature.

[31] Hagège H, Klous P, Braem C, Splinter E, Dekker J, Cathala G, de Laat W, Forné T (2007). Quantitative analysis of chromosome conformation capture assays (3C-qPCR).. Nat Protocol.

