# Supplementary figures and images for: Active RNA Polymerases: Mobile or Immobile Molecular Machines?
Source: PLoS Biol. 2010 Jul 13;8(7):e1000419. doi: 10.1371/journal.pbio.1000419 (PMC2903595; doi:10.1371/journal.pbio.1000419)

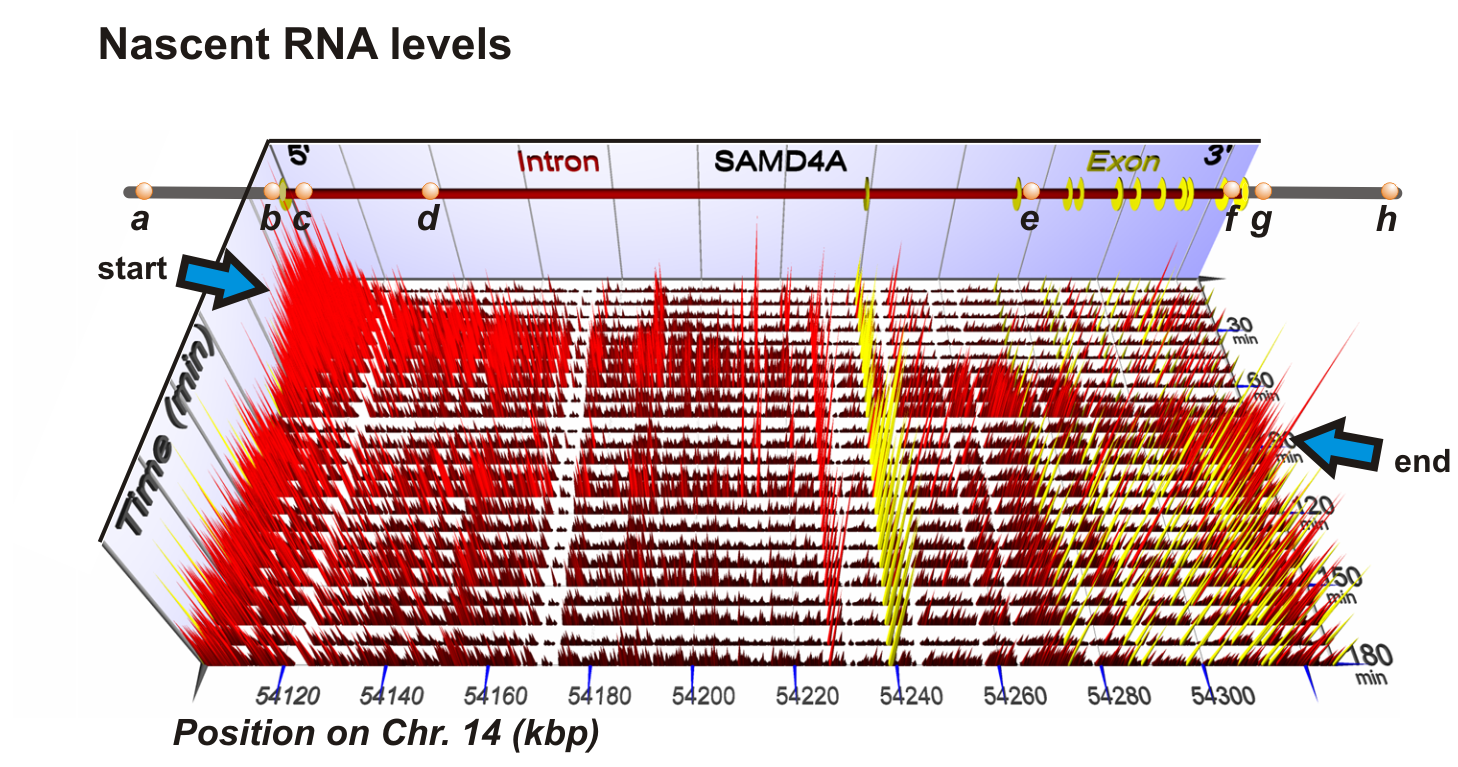

Supplement: Figure S1 — TNFα induces a wave of transcription to sweep along SAMD4A . HUVECs were treated with TNFα, samples collected every 7.5 min for 3 h, total RNA purified and hybridized to a tiling microarray bearing 25-mers complementary to SAMD4A (modified from [9]). On the gene map (top) positions of introns, exons, and targets of 3C primers a–h are indicated. Position a corresponds to 25 kbp 5′ before the transcription start site (tss), b to the promoter, c to the beginning of intron 1, d to 34 kbp into intron 1, e to intron 3, f to intron 11, g to the 3′ untranslated region (utr), and h to 25 kbp after the poly(A) site. The vertical axis gives intensity of signal of intronic and exonic probes (red and yellow vertical needles, respectively); genomic location (bottom) and time after stimulation (top to bottom) are shown. No transcripts copied from either sense or anti-sense strands are detected at 7.5 min [9]. A wave of signal initiates at the 5′ end within 15 min (start), and then travels down the gene to terminate after 75–90 min (end). Co-transcriptional splicing and premature termination conspire to generate this wave (e.g., as the wave reaches the middle of intron 2 after 60–75 min, little signal is seen in intron 1). Note also that probes covering the first thousands of nucleotides from the tss yield signal between 15–180 min, and polymerases only seem to escape downstream in a limited interval (i.e., after 15–30 min) to initiate a first, fairly synchronous wave. This points to a checkpoint regulating escape; it seems to act on a second polymerase once it senses there is already a first on the gene (despite being perhaps 100 kbp downstream). This figure is reproduced from [9]. (1.27 MB TIF) [file pbio.1000419.s001.tif]

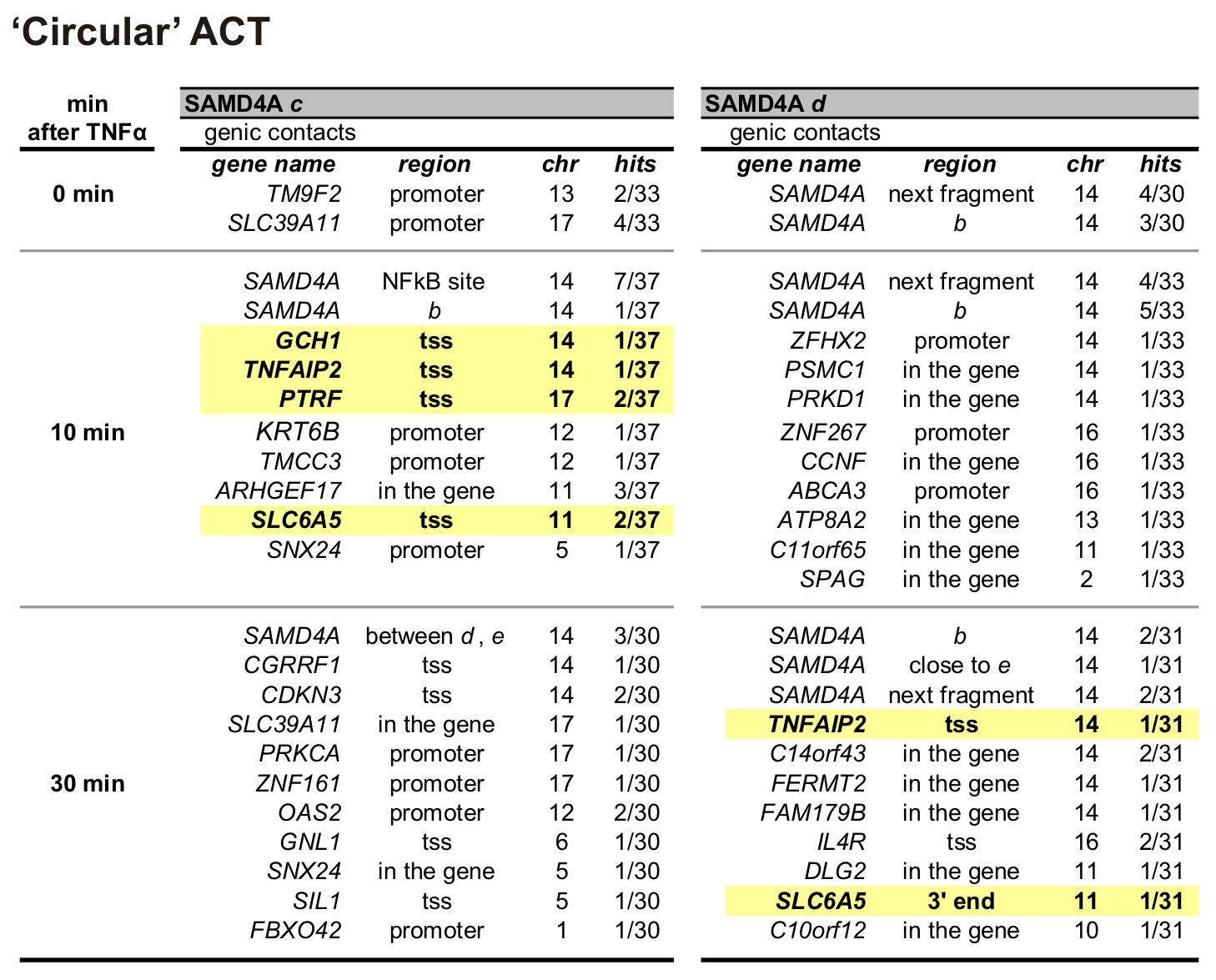

Supplement: Figure S2 — Changing contacts detected using “circular ACT” (associated chromosome trap). To detect intra-/inter-chromosomal contacts made by SAMD4A regions c and d at 0, 10, and 30 min after adding TNFα, we performed circular ACT [13],[14]. 3C templates were prepared using either SacI or HindIII and then Csp6I, nested inverse PCR conducted (using primers targeting SAMD4A regions c or d), products cloned and sequenced, and segments contacting SAMD4A mapped. Genic contacts with gene name, region of gene, chromosomal location, and the number of times (hits) that particular sequence was seen compared to the total number of sequences analyzed (includes self-ligation products and contacts with non-coding regions that are not shown) are listed. Results support the idea that, at 0 min, SAMD4A makes few contacts. After 10 min, region c contacts many more genes, including partners (highlighted) we study (TNFAIP2, GCH1, SLC6A5, PTRF); no such contacts are seen with region d (the wave of transcription has not yet reached this region). After 30 min, region d now contacts TNFAIP2 and SLC6A5 (in accord with 3C data in Figures 3 and 4; note a contact between SAMD4A and the tss of SLC6A5 is detected at 10 min, and one with the 3′ end of SLC6A5 at 30 min). In a population of cells, a gene contacts other genomic regions with varying frequencies [26],[27], and circular ACT detects those occurring the most often (to give repeated “hits” in independent experiments) against an inevitable background [13],[14]. As in independent experiments we detect contacts between SAMD4A and TNFAIP2, SLC6A5, PTRF1 (shown here), and GCH1 (one contact shown here, plus one additional one seen after 60 min; not shown), it is likely that all these interactions are major ones—although not necessarily the strongest ones. (0.41 MB TIF) [file pbio.1000419.s002.tif]

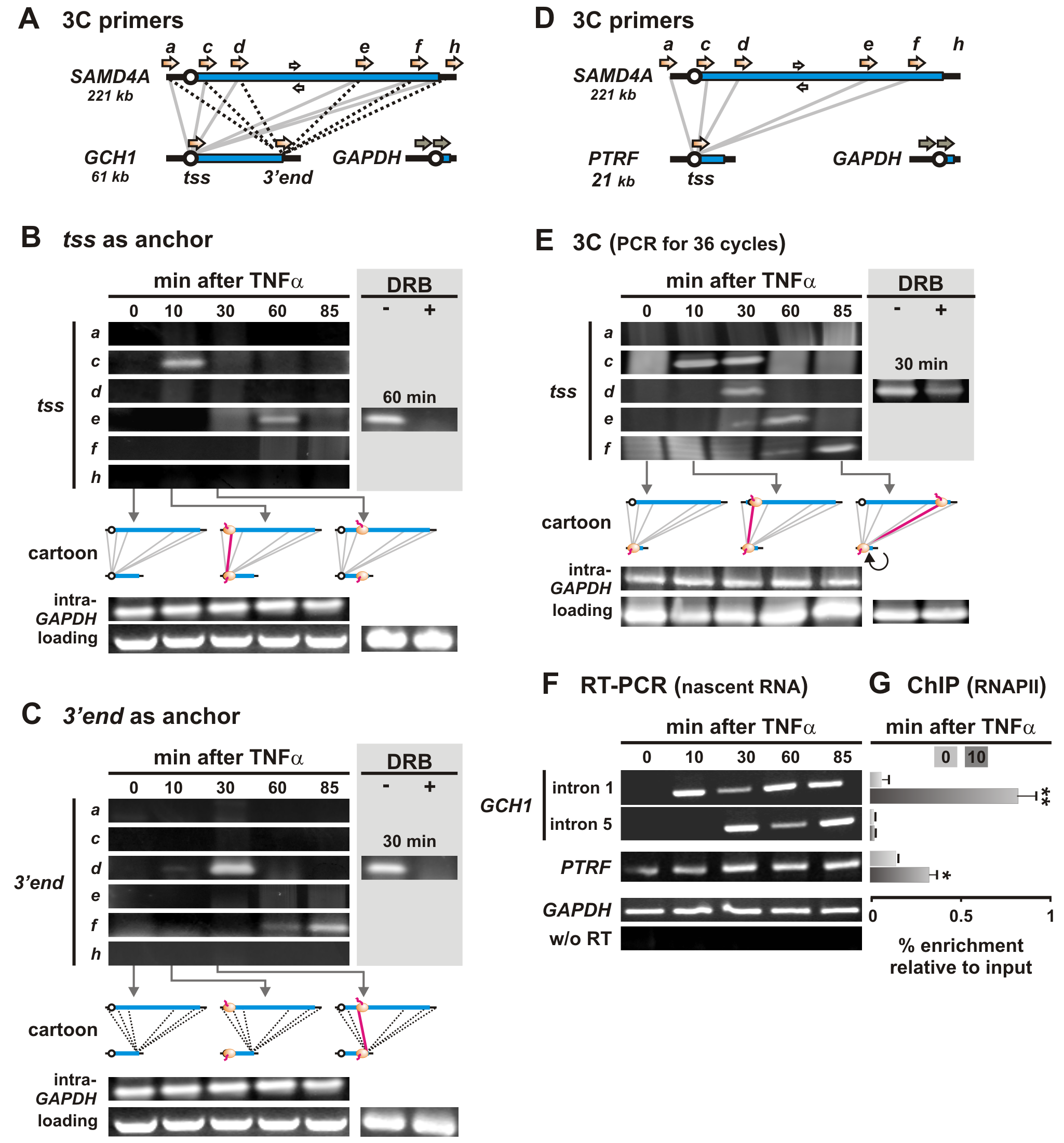

Supplement: Figure S3 — Contacts between SAMD4A and GCH1 (or PTRF ) follow engaged polymerases. General details are as in Figure 3A. (A) Positions of 3C primers targeting SAMD4A and GCH1, which lie ∼0.8 Mbp apart on chromosome 14. (B, C) Contacts between SAMD4A and the 5′ and 3′ ends of GCH1. The interaction pattern is similar to that seen with SAMD4A and SLC6A5 (which is of comparable length to GCH1; Figure 4). Panel (B) shares with (C) the same intra-GAPDH and loading controls (excluding ± DRB). (D) Positions of 3C primers targeting SAMD4A and PTRF. (E) Contacts between SAMD4A and the tss of PTRF (on chromosome 17). The interaction pattern is similar to that seen between SAMD4A and TNFAIP2 (Figure 3). (F) Nascent RNA detected by RT-PCR in total RNA isolated from HUVECs 0–85 min after adding TNFα. For GCH1 at 0 min, no signal is seen. After 10 min, maximal levels of RNA are seen at the tss (intron 1); after 30 min, they are seen at the 3′ end (intron 5). This cycle repeats between 60 and 85 min. PTRF is expressed prior to TNFα induction, but levels of intronic RNA increase after stimulation. Controls show that levels of GAPDH intronic RNA remain unchanged and that amplimers do not result from contaminating genomic DNA (w/o RT). (G) Levels of bound RNA polymerase II (detected by ChIP using anti-phospho-Ser5 in the C-terminal domain of the largest subunit) 0–10 min after stimulation (light and dark grey bars, respectively). Levels of enrichment are expressed relative to those of the input; values for different amplicons are normalised relative to those seen with GAPDH. Error bars show standard deviations from two independent experiments. *p<0.05, **p<0.01, Student's t test compared to 0 min. (1.11 MB TIF) [file pbio.1000419.s003.tif]

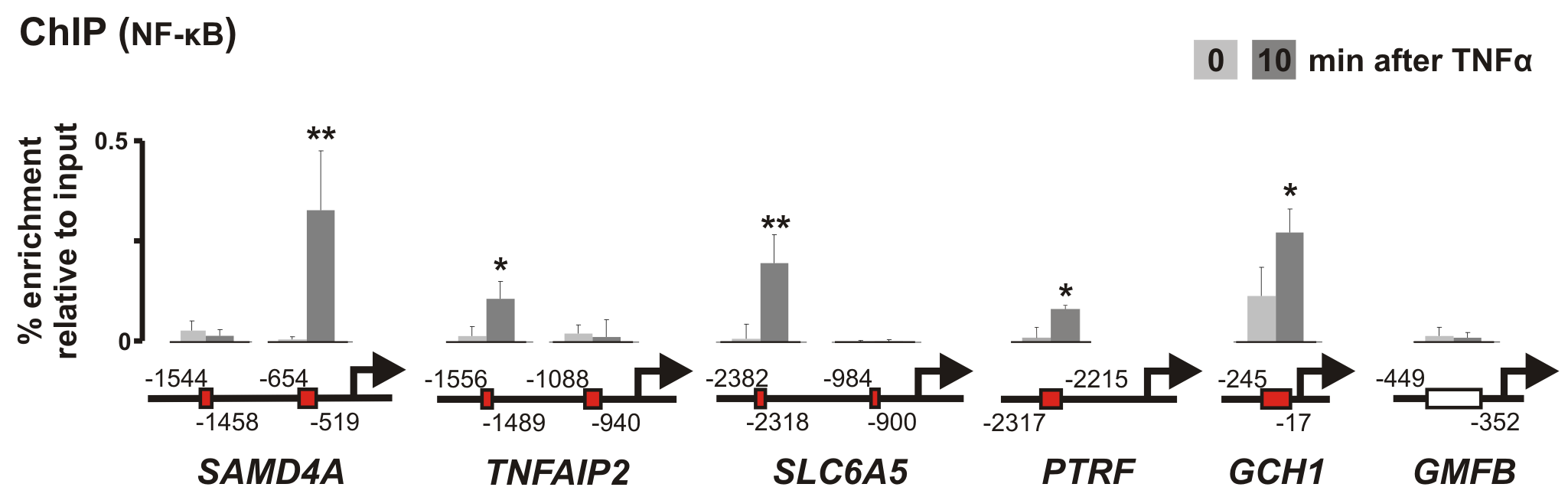

Supplement: Figure S4 — NF-κB binds to promoters of TNFα-responding genes within 10 min. HUVECs were treated with TNFα, and binding of NF-κB (p65 subunit) assessed by ChIP using chromatin obtained 0–10 min (light and dark grey bars, respectively) post-induction. Putative NF-κB binding sites (5′-GGGRNNYCC-3′; red boxes) in the 5′ proximal regions of five genes are indicated; the GMFB promoter region (white box) contains no such sites and serves as a negative control. Bars over each targeted region show the percentage enrichment relative to input DNA. Error bars show standard deviations from three independent experiments. *p<0.05, **p<0.01, Student's t test compared to 0 min. (0.19 MB TIF) [file pbio.1000419.s004.tif]

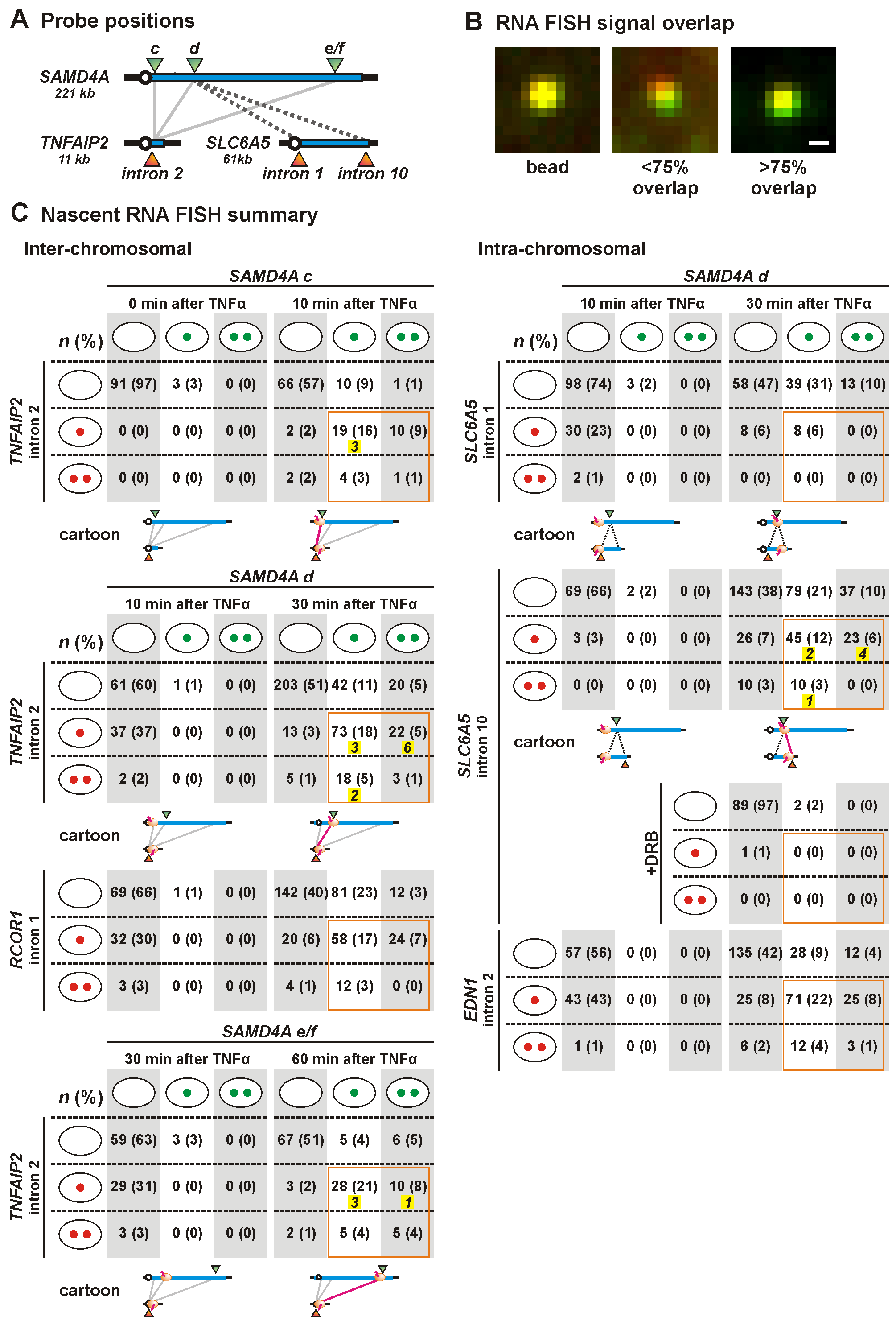

Supplement: Figure S5 — Summary of RNA FISH results. (A) Positions of RNA FISH probes that target introns within SAMD4A (green triangles), TNFAIP2, and SLC6A5 (red triangles). (B) Criteria used to assess overlap of red and green foci. The image on the left provides a colocalizing control: a 110 nm bead that fluoresces in both red and green channels to give yellow in this merged image. The images in the middle and on the right are of foci collected as in Figure 5F using probes targeting SAMD4A region d (green) and SLC6A5 intron 5 (red) 30 min after induction. A focus is defined as >4 contiguous (90 nm) pixels that contain signal above a threshold (defined as the average intensity of at least 50 pixels in a line-scan across the focus); typically, foci were 12±4 pixels in size and were classified as red or green (no signal of the other colour above threshold in >75% pixels) or yellow (signal above threshold of both colours in ≥75% pixels). The middle image is therefore scored as one red and one green focus even though the two partially overlap; such partially overlapping foci were rare (constituting <3% of all foci). The image on the right is scored as a yellow focus (as >75% pixels in the focus contain both green and red signals above the threshold). Bar: 200 nm. (C) Summary of RNA FISH results. HUVECs were treated with TNFα for 10–60 min, RNA FISH performed with probe pairs detecting nascent RNA copied from the regions indicated, and numbers of cells containing red, green, and yellow foci determined (from images like those in Figure 5A–G). In each case, one probe (green) targets RNA copied from regions c, d, or e/f of SAMD4A, while a second (red) targets intronic RNA from either a control gene that yields no 3C product with SAMD4A (i.e., RCOR1, EDN1) or a test gene (i.e., TNFAIP2, SLC6A5) that does. Values represent numbers of cells (n) with the patterns indicated (percentages in brackets); numbers of yellow foci are highlighted. A probe targeting the anti-sense strand of SAMD4A region d, a [file pbio.1000419.s005.tif]

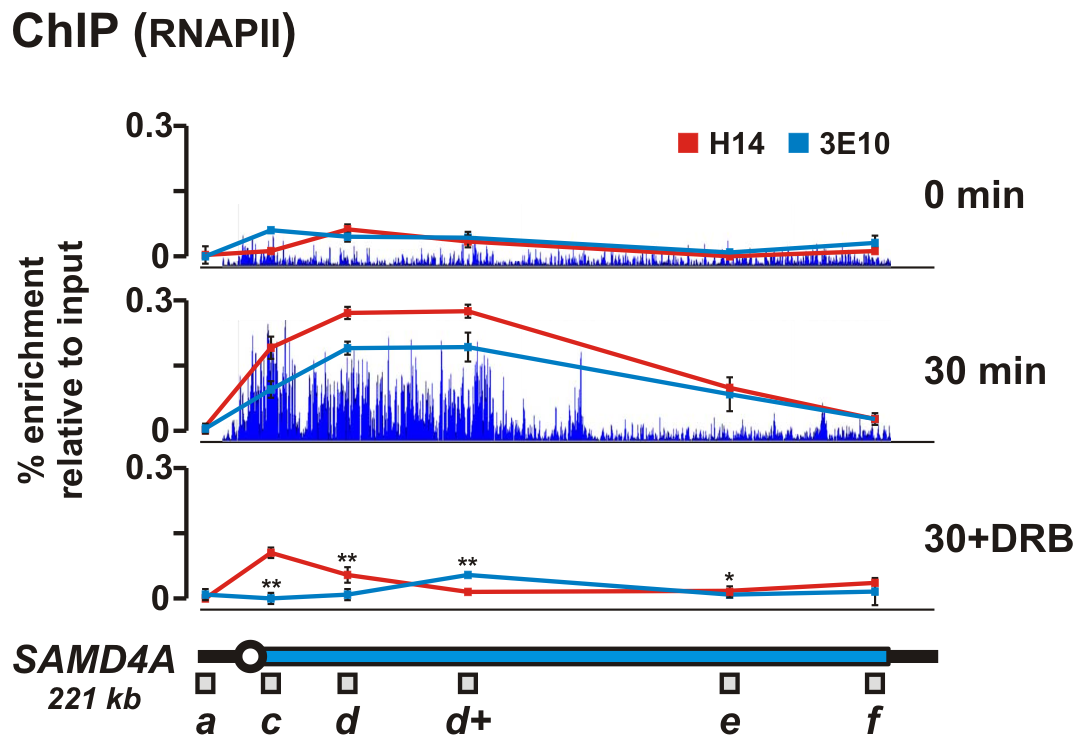

Supplement: Figure S6 — Binding of phosphorylated forms of RNA polymerase II along SAMD4A . ChIP was performed using antisera predominantly recognizing the largest subunit of RNA polymerase II phosphorylated at serine 5 (H14; red curves) or serine 2 (3E10; blue curve) in the heptad repeats of the C-terminal domain [18]. The cartoon below indicates probe positions. Chromatin was isolated from HUVECs 0 or 30 min after induction; in some cases DRB was added 20 min before harvesting. For the first two panels, ChIP-chip results (blue) adapted from [9] are included. At 0 min, little signal is seen along the gene; at 30 min, significant amounts of the polymerase are bound on the first third of the gene. Upon DRB treatment (bottom panel), phospho-serine 2 signal returns to background levels, whereas phospho-serine 5 signal accumulates around the tss, as might be expected [17],[18]. Experiments were performed on two independently prepared templates; error bars show standard deviations (*p<0.05, **p<0.01, Student's t test compared to 30 min). (2.44 MB TIF) [file pbio.1000419.s006.tif]

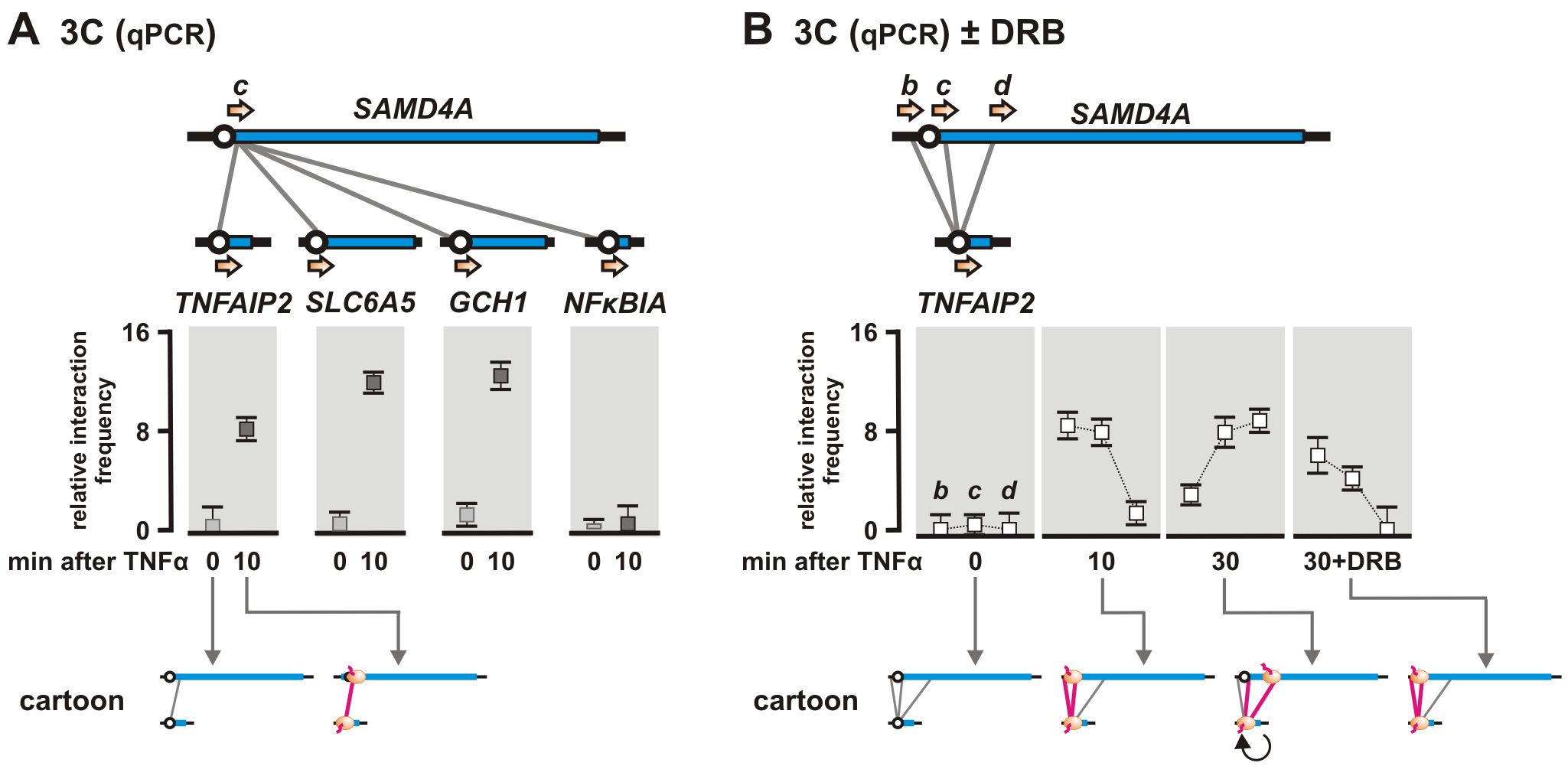

Supplement: Figure S7 — Selected 3C interactions assessed by quantitative real-time PCR. HUVECs were treated with TNFα for 0–30 min, 3C templates prepared using SacI, and qPCR conducted using equal weights of DNA and primers targeting indicated regions; amounts of 3C products detected were normalized relative to intra-GAPDH 3C amplimers (as in [27]). In some cases DRB was added 20 min before harvesting cells. Cartoons illustrate where polymerases are bound at different times and the interactions analyzed (grey lines); red lines indicate interactions detected, and these correlate with the presence of a polymerase on both partners. Values are averages (± standard deviation) from three independent experiments. (A) Interactions between SAMD4A fragment c and four TNFα-responsive genes. Strong interactions are seen with three genes (but not NFKBIA). (B) Interactions between SAMD4A fragments b–d and TNFAIP2. Strong interactions are seen at appropriate times, confirming results in Figure 3. As DRB inhibits productive elongation (see Figure S6), interactions around the promoter and tss are still detected. (0.32 MB TIF) [file pbio.1000419.s007.tif]

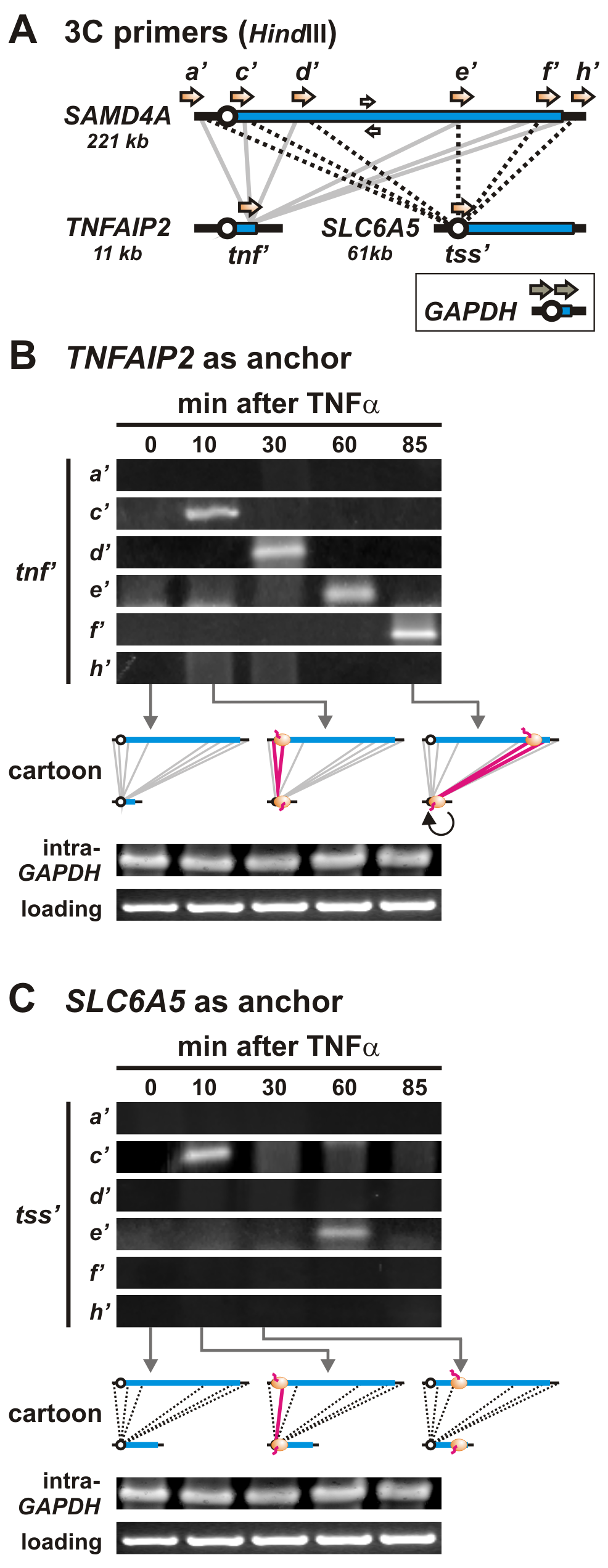

Supplement: Figure S8 — Using Hind III to prepare 3C templates yields interactions like those seen with Sac I. Details are as for Figures 3 and 4; essentially the same changing patterns are detected. (A) Positions of 3C primers. (B) Interactions between SAMD4A and TNFAIP2. (C) Interactions between SAMD4A and the tss of SLC6A5. (0.63 MB TIF) [file pbio.1000419.s008.tif]

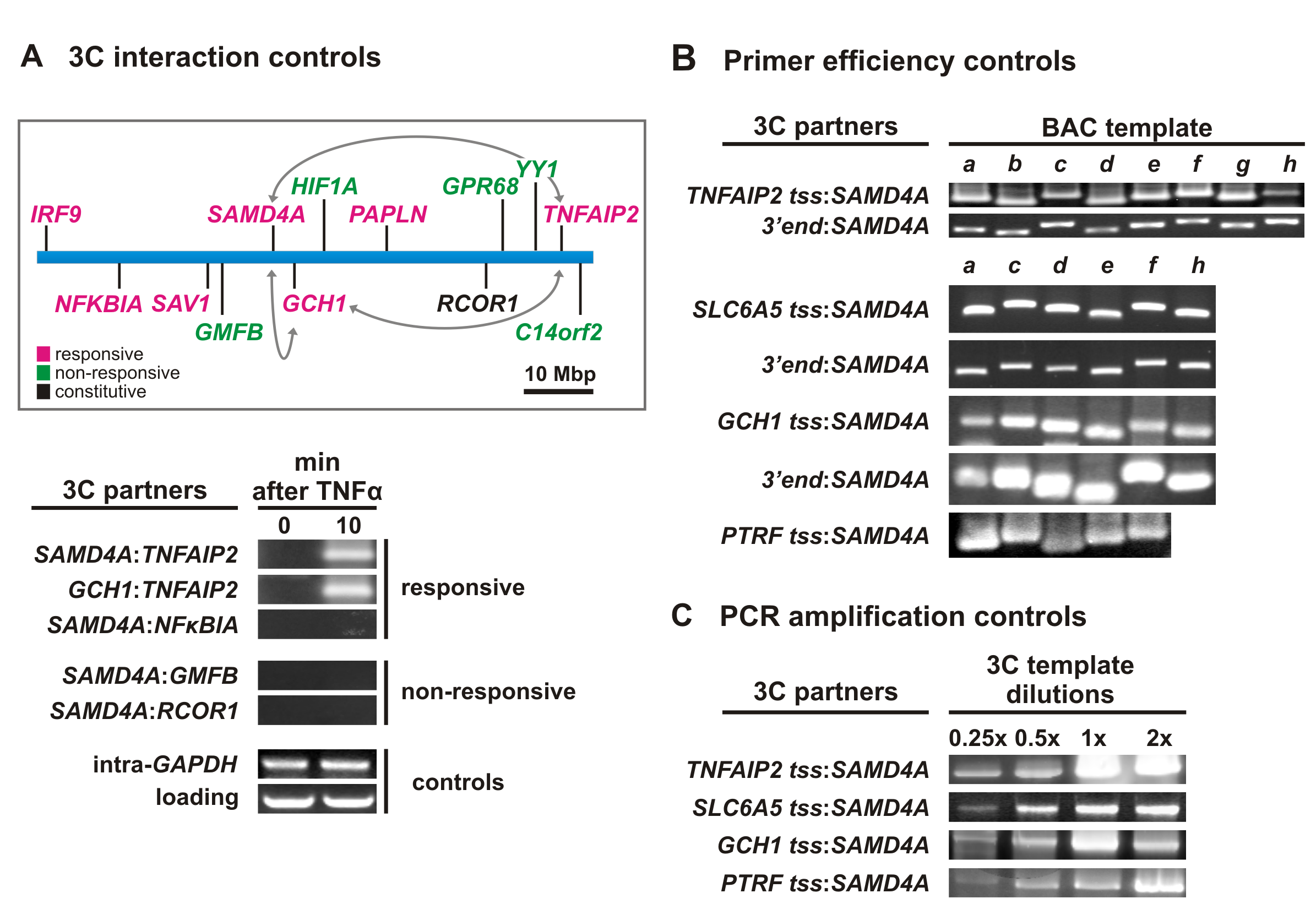

Supplement: Figure S9 — Some 3C controls. (A) Specificity of inter-genic interactions. Genes screened (TNFα-responsive, non-responsive, and constitutive) are indicated on the map of part of human chromosome 14 (from genome reference assembly 37). HUVECs were treated with TNFα for 0–10 min, 3C templates prepared using SacI, and PCR conducted using primers targeting the tss of each gene. SAMD4A contacts the TNFα-responsive gene TNFAIP2 (grey arrow) which lies ∼50 Mbp downstream, but not another responding gene—NFKBIA—lying ∼20 Mbp upstream, nor two non-responsive genes—GMFB, RCOR1—lying ∼0.1 and ∼40 Mbp downstream. [Additional responding non-interactors included SAV1, IRF1, GPR68, and PAPLN; additional non-responding non-interactors included YY1, HIF1A, C14orf2 (not shown).] Responsive genes GCH1 and TNFAIP2 also contact one another (grey arrow). 3C products obtained from two parts of GAPDH yield uniform levels of amplimers, as do loading controls. (B) Controls for amplification efficiencies of primers. Amplification efficiencies were assessed using a control template generated by digestion of BAC clones with SacI followed by ligation. As in (A), PCR was conducted using equal weights of these templates and primers targeting regions indicated. Different primer pairs yield comparable amounts of amplimers. (C) 3C conducted using serial 2-fold dilutions of template to assess the range of linear amplification. In the examples shown, 3C templates are derived from HUVECs treated with TNFα for 10 min; 1× dilution represents 200 ng of template per 25 µl reaction volume. 3C reactions shown in all other figures were adjusted accordingly. (0.97 MB TIF) [file pbio.1000419.s009.tif]
