# Supplementary material for: Active RNA Polymerases: Mobile or Immobile Molecular Machines?
Source: PLoS Biol. 2010 Jul 13;8(7):e1000419. doi: 10.1371/journal.pbio.1000419 (PMC2903595; doi:10.1371/journal.pbio.1000419)
Supplement: Text S1 — Supplementary information. Detailed Materials and Methods. (0.05 MB DOC) [file pbio.1000419.s010.doc]

**Active RNA Polymerases: Mobile or Immobile Molecular Machines?**

Argyris Papantonis, Joshua D. Larkin, Youichiro Wada, Yoshihiro Ohta, Sigeo Ihara,

Tatsuhiko Kodama, and Peter R. Cook

**Supporting Information**

- Supporting Materials and Methods

- References in SI

**Supporting Materials and methods**

*Oligonucleotides*

All PCR primers were designed using Primer 3.0 Plus (<http://www.bioinformatics.nl/cgi-bin/primer3plus/primer3plus.cgi>) to have an optimal length of 22 nucleotides, a melting temperature of 62°C, and to yield amplimers of 100-200 bp. Primer sequences are available upon request.

*Reverse transcriptase PCR (RT-PCR)*

Total RNA was isolated from 10^7^ cells using TRIzol LS (Invitrogen), treated with RQ1 DNase (Promega), and nascent RNA amplified using the One-Step RT-PCR kit (Invitrogen) with primers targeting introns; amplimers were then resolved in 1.5% agarose gels, stained with SYBR Green nucleic acid stain I (Invitrogen), imaged, and their relative levels assessed using an FLA-5000 laser scanner and AIDA software (Fuji). Side-by-side reactions in which Platinum *Taq* polymerase (Invitrogen) replaced the reverse transcriptase/*Taq* polymerase mix were performed to ensure amplimers did not result from residual genomic DNA.

*Circular associated chromosome trap*

This approach was based on “associated chromosome trap” (ACT) [13] and 3C-inverse PCR [14]. Approximately 1 μg of 3C template (prepared from cells 10 min after TNFα induction, using *Sac*I; above) was cut with *Csp*6I (Invitrogen), self-ligated in 1 ml (at ~0.5 ng/μl; 72 h; 4°C) using 400 units of T4 DNA ligase, and purified using PCR purification columns (Qiagen); 1 μl (1/50^th^ of the eluate) was then used in a nested inverse PCR (first primer pair – 95°C for 2 min, plus 16 cycles at 95°C for 55 sec, 58°C for 45 sec, and 72°C for 30 sec, followed by one cycle at 72°C for 3 min; second primer pair – 95°C for 2 min, 22 cycles at 95°C for 55 sec, 60°C for 35 sec, and 72°C for 25 sec, followed by one cycle at 72°C for 2 min). Amplimers were resolved on 1.6% agarose gels, bands excised, DNA purified using a gel extraction kit (Qiagen) and cloned into the pGEM-T vector (Promega) according to the manufacturer’s instructions; TOP10 cells (Invitrogen) were transformed, plated, and plasmid inserts from all resulting colonies sequenced. Results from two independent experiments are shown in Fig. S2. To control for random amplification, the procedure was also run on non-digested/ligated and digested/non-ligated 3C templates, which yielded no amplimers.

*RNA fluorescent in situ hybridization (RNA FISH)*

RNA FISH was performed essentially as described by Wada *et al*. [9], using three types of probes: (i) Sets of five 50-mer probes (Gene Design, Japan); two targeting *SAMD4A* region *d* (~34 kbp into intron 1, either sense or anti-sense strands), one against region *c* (~1.5 kbp into intron 1), one against region *e/f* (in intron 7), and one targeting *EDN1* intron 2. In each 50-mer, roughly every tenth thymine residue was substituted by an amino-modifier C6-dT. The amino group was subsequently tagged with Alexa Fluor 488 or 647 reactive dye (Invitrogen) or Cy3 Mono Reactive Dye (GE Healthcare) according to the manufacturers’ instructions. Probes were then purified using G-50 columns (GE Healthcare), ethanol precipitated twice, and concentrated using a Microcon-30 column (Millipore). Labeling efficiencies were calculated using the Base:Dye ratio calculator (Invitrogen; <http://probes.invitrogen.com/resources/calc/basedyeratio.html>), and were between 7-9 fluors per 100 nucleotides. (ii) A set of 36 20-mer probes (Biosearch), targeting either *SLC6A5* (introns 1, or 10) or *RCOR1* (intron 1). Each 20-mer bore a 3’-amino-modification to allow labeling with Alexa 488, 647 or Cy3 as described above. Labeling efficiencies (calculated as above) were between 4.5-5 fluors per 100 nucleotides. (iii) A nick-translated probe targeting the second intron of *TNFAIP2* was amplified by PCR and labeled with digoxygenin (DIG) by nick translation using a kit (Roche); the hybridized probe was detected by indirect immunolabeling using a primary sheep anti-DIG (1/_250_ dilution, 1-333-089; Roche) and a secondary anti-sheep IgG Cy3- or Cy5-conjugated antibody (1/_500_ dilution, 713-165-147; Jackson ImmunoResearch). For each experiment, HUVECs on coverslips were grown to 65-75% confluency, treated (0-60 min) with TNFα, fixed (15 min; room temperature) in 4% paraformaldehyde/0.05% acetic acid/0.15 M NaCl, washed 3 times in PBS, permeabilized (5 min; 37°C) in 0.01% pepsin (pH 2.0), rinsed in water treated with diethyl-pyrocarbonate, postfixed (5 min; room temperature) in 4% paraformaldehyde/PBS, and stored overnight at -20°C in 70% ethanol. Coverslips were dehydrated in 70%, 80%, 90% and 100% ethanol, placed at 37°C in a moist chamber, and hybridized overnight with 25 ng labelled probes in 25% deionized formamide, 2x SSC, 250 ng/ml sheared salmon sperm DNA, 5x “Denhardt’s” solution, 50 mM phosphate buffer (20 mM KH_2_PO_4_, 30 mM KHPO_4_·2H_2_O, pH 7.0) and 1 mM EDTA. [SSC is 0.15 M NaCl and 0.015 M sodium citrate; 50x Denhardt’s solution contains 1% Ficoll 400, 1% polyvinylpyrolidone and 1% bovine serum albumin in water treated with diethylpyrocarbonate.] Next, cells were washed once in 4x SSC (15 min; 37°C), twice in 2x SSC (5 min; 37°C), twice in 2x SSC/25% formamide (5 min; 37°C), once in 2xSSC (5 min; 37°C) and mounted in Vectashield (Vector Laboratories) complemented with 1 µg/ml DAPI (4,6-diamidino-2-phenylindole; Sigma) and fluorescent reference beads (Invitrogen; used as internal normalization controls to correct for day-to-day variations in the microscope). In cases where antibody detection was used, the following steps were incorporated after hybridization: wash twice (5 min; 20°C) in Tris-saline-Tween (0.15 M NaCl, 0.1 M Tris·HCl, 0.05% Tween-20, pH 7.5), block (30 min; 20°C) in 1.35% blocking reagent (Roche) in Tris-saline (0.15 M NaCl, 0.1 M Tris·HCl, pH 7.5), rinse twice (5 min; 20°C) in Tris-saline-Tween, add primary antibody (30 min; 20°C), wash twice in Tris-saline-Tween, add secondary antibody (30 min; 20°C), and wash (5 min; 20°C) in Tris-saline. Images were collected on an Axioplan 2 or an Axiovert microscope (Zeiss) equipped with a CCD camera (CoolSNAP_HQ_, Photometrics) and analyzed using MetaMorph v. 7.0 (Molecular Devices). The contrast of pictures shown was adjusted to fit the greyscale.

*Localization of foci with sub-diffraction resolution and modeling of their spacing*

The relative distance between overlapping red and green foci was measured using a common method for localizing sub-resolution particles [21-23]. First, the position of each focus in a pair was established relative to the image frame, and the Euclidean distance between the two measured. A 2D Gaussian intensity profile was statistically fit to an image of a focus using regression analysis to minimize least-squares distances between intensity values. Foci were assumed to be smaller than the diffraction limit of light, and the center of a focus was determined to be at the position of the maximum of the Gaussian profile. Uncertainty about the position of a focus was estimated based on imaging parameters [23]. To correct for pixel shift between fluorescence channels, the distance between images of the same red-green fluorescent bead (110 nm TetraSpeck™ microspheres, Invitrogen) was measured. The difference in alignment was accounted for in calculations of localization uncertainty.

The observed distribution of lateral distances between red and green foci in Figure 5H is consistent with the underlying transcripts being randomly positioned on the surface of ~87-nm factories [20]. Therefore we modeled the system as a set of randomly-distributed points confined to a shell of 5, 15, 25, or 35 nm around an 87-nm sphere, projected this 3D model on to a 2D plane, and measured the lateral distances between all pairs of points; the best fit with experimental data was obtained with a 35-nm shell (normal distribution). All calculations were performed in MATLAB (The MathWorks, Inc.) using custom software (available on request).

*Chromatin immunoprecipitation (ChIP)*

Approximately 10^7^ HUVECs were crosslinked (10 min; room temperature) in 1% paraformaldehyde at the appropriate time. Chromatin was prepared, fragmented, washed and eluted using the ChIP-It-Express kit following instructions for enzymatic shearing (Active motif). Immunoprecipitations were performed using a polyclonal against the NF-κB p65 subunit (1 μl/reaction; 2 μg/μl; 06-418 from Upstate) and three different monoclonals against the heptads in the C-terminal domain of the largest subunit of RNA polymerase II – (i) in Figures 2 and S3G, a mouse monoclonal (Pa57) against phospho-Ser5 (10.3 μg/μl; a gift of H. Kimura), (ii) in Figure S6, a mouse monoclonal (H14), also against phospho-Ser5 (2 μg/μl; MMS-134R from Covance; [9]), and (iii) in Figure S6, a rat monoclonal (3E10) against phospho-Ser2 (10.8 μg/μl; a gift of D. Eick; [18]). DNA was purified using a PCR clean-up kit (Qiagen) prior to quantitative real-time PCR.

*Quantitative real-time PCR (qPCR)*

For both 3C and ChIP, qPCR was performed using a Rotor-Gene 3000 cycler (Corbett) and Platinum SYBR Green qPCR SuperMix-UDG (Invitrogen). Following incubation at 50°C for 5 min to activate the PCR mix, and 95°C for 4 min to denature templates, reactions were for 40 cycles at 95°C for 15 sec, and 60°C for 50 sec. The presence of single amplimers was confirmed by melting curve analysis and gel electrophoresis, and quantitation performed using Rotor-Gene software (Corbett). Data was analyzed as described [31,32].

**References in Supporting Information**

32. Nelson JD, Denisenko O, Bomsztyk K (2006) Protocol for the fast chromatin immunoprecipitation (ChIP) method. Nat Protoc 1: 179-185.
